# Intestinal transit-amplifying cells require METTL3 for growth factor signaling and cell survival

**DOI:** 10.1172/jci.insight.171657

**Published:** 2023-12-08

**Authors:** Charles H. Danan, Kaitlyn E. Naughton, Katharina E. Hayer, Sangeevan Vellappan, Emily A. McMillan, Yusen Zhou, Rina Matsuda, Shaneice K. Nettleford, Kay Katada, Louis R. Parham, Xianghui Ma, Afrah Chowdhury, Benjamin J. Wilkins, Premal Shah, Matthew D. Weitzman, Kathryn E. Hamilton

**Affiliations:** 1 Division of Gastroenterology, Hepatology, and Nutrition, Department of Pediatrics, Children’s Hospital of Philadelphia, Perelman School of Medicine;; 2 Medical Scientist Training Program, Perelman School of Medicine; and; 3 Perelman School of Medicine, University of Pennsylvania, Philadelphia, Pennsylvania, USA.; 4 Department of Biomedical and Health Informatics, Children’s Hospital of Philadelphia, Philadelphia, Pennsylvania, USA.; 5 Division of Protective Immunity, Children’s Hospital of Philadelphia Research Institute, Philadelphia, Pennsylvania, USA.; 6 Department of Pathology and Laboratory Medicine, Perelman School of Medicine; University of Pennsylvania, Philadelphia, Pennsylvania, USA.; 7 Waksman Institute of Microbiology and; 8 Department of Genetics, Rutgers University, Piscataway, New Jersey, USA.; 9 Human Genetics Institute of New Jersey, Piscataway, New Jersey, USA.; 10 Department of Pathobiology, School of Veterinary Medicine, and; 11 Institute for Regenerative Medicine, University of Pennsylvania, Philadelphia, Pennsylvania, USA.

**Keywords:** Gastroenterology, Adult stem cells, Homeostasis, RNA processing

## Abstract

Intestinal epithelial transit-amplifying cells are essential stem progenitors required for intestinal homeostasis, but their rapid proliferation renders them vulnerable to DNA damage from radiation and chemotherapy. Despite these cells’ critical roles in intestinal homeostasis and disease, few studies have described genes that are essential to transit-amplifying cell function. We report that RNA methyltransferase-like 3 (METTL3) is required for survival of transit-amplifying cells in the murine small intestine. Transit-amplifying cell death after METTL3 deletion was associated with crypt and villus atrophy, loss of absorptive enterocytes, and uniform wasting and death in METTL3-depleted mice. Sequencing of polysome-bound and methylated RNAs in enteroids and in vivo demonstrated decreased translation of hundreds of methylated transcripts after METTL3 deletion, particularly transcripts involved in growth factor signal transduction such as *Kras*. Further investigation verified a relationship between METTL3 and *Kras* methylation and protein levels in vivo. Our study identifies METTL3 as an essential factor supporting the homeostasis of small intestinal tissue via direct maintenance of transit-amplifying cell survival. We highlight the crucial role of RNA modifications in regulating growth factor signaling in the intestine with important implications for both homeostatic tissue renewal and epithelial regeneration.

## Graphical abstract

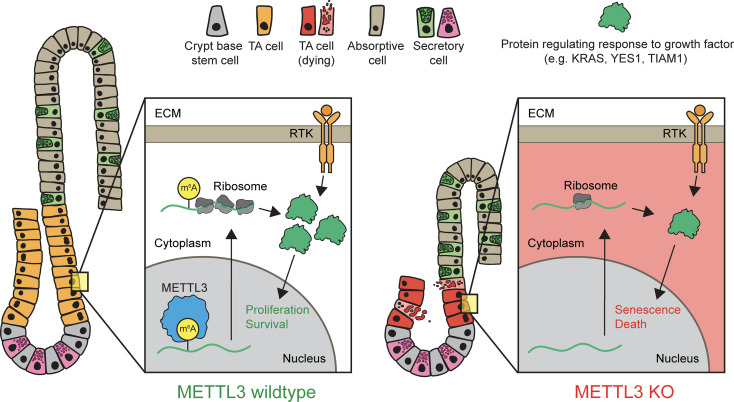

## Introduction

The intestinal epithelium digests and absorbs nutrients, protects against pathogen invasion, and regulates interactions between mucosal immune cells and the gut lumen (1). These essential functions are made possible by the continuous renewal of differentiated intestinal epithelial cells (2). Epithelial renewal begins at the intestinal crypt base, where intestinal stem cells expressing the Wnt target gene, leucine-rich repeat-containing G protein–coupled receptor 5 (LGR5), initiate differentiation and migrate up the crypt wall. As they move up the crypt wall, LGR5^+^ stem cells differentiate into intestinal stem progenitors known as transit-amplifying (TA) cells (2). TA cells rapidly undergo successive cycles of proliferation to generate the bulk of intestinal epithelium. While existing research emphasizes the role of LGR5^+^ stem cells in tissue renewal, TA cells are the primary site of intestinal epithelial proliferation and differentiation, and they produce the majority of differentiated epithelium (3, 4). Rapid proliferation is the central defining feature of TA cells, and it renders them particularly vulnerable to DNA-damaging agents such as chemotherapeutics and radiation therapy (5–7). These routine cancer treatments cause chemotherapy-induced gastrointestinal toxicity (CIGT) and radiation-induced gastrointestinal syndrome (GIS), which together affect more than 80% of patients with cancer. CIGT and GIS are debilitating pathologies with limited treatment options (8–10). One potential therapeutic avenue would be the development of drugs that protect the TA cells preferentially damaged by these common cancer therapies. However, despite TA cells’ critical roles in intestinal homeostasis and disease, factors that maintain survival and proliferation of TA cells remain inadequately defined compared with the extensive study of LGR5^+^ stem cells.

Novel approaches are needed to identify factors that specifically regulate TA cell function. While numerous studies have defined transcriptional control of intestinal stem cells, posttranscriptional regulation of intestinal epithelial homeostasis is only beginning to be understood. Recent research points to important roles for the RNA modification, N^6^-methyladenosine (m^6^A), in intestinal crypts (11–15). m^6^A is the most common covalent modification of RNA, occurring on approximately 25% of mRNA transcripts (16, 17). It acts by recruiting RNA-binding proteins that affect mRNA fate, predominantly stability and translation. (18). Global m^6^A methylation patterns in the epithelium can shift with microbial and nutrient contents of the gut, and m^6^A-binding proteins have been implicated in intestinal regeneration and the pathogenesis of inflammatory bowel disease (11, 13, 15, 19, 20). These studies suggest critical roles for m^6^A in integrating environmental cues with homeostatic and regenerative processes in the intestinal epithelium.

Despite advances in the study of m^6^A in the gut, the effect of global depletion of m^6^A in the intestinal epithelium remains unclear and incomplete, particularly in the small intestine. A highly conserved m^6^A “writer” complex installs m^6^A cotranscriptionally in the nucleus of eukaryotic cells. At the core of this complex are the writer proteins methyltransferase-like 3 and 14 (METTL3 and METTL14) (18). Although METTL3 is the catalytic subunit, both METTL3 and METTL14 are thought to be essential for the methylating activity of the complex (21, 22), and both METTL proteins are deleted interchangeably to define the role of m^6^A in specific tissue or cell types. Recent studies reported essential functions for METTL14 in the survival of LGR5^+^ stem cells in the colon, with complete sparing of the small intestine (23, 24). In contrast, another very recent study found that METTL3 deletion caused defects in LGR5^+^ stem cells in the small intestine (25). However, in the case of METTL3 deletion, rescue of LGR5^+^ stem cell survival could not rescue tissue homeostasis. Therefore, the critical defect in METTL3-depleted epithelium remains unclear.

In contrast with previous reports emphasizing dysfunction of LGR5^+^ stem cells, we found that METTL3 deletion induced profound cell death predominantly in small intestinal TA cells. Disruption of the TA zone was associated with crypt and villus atrophy and widespread reduction in absorptive enterocytes, ultimately resulting in the death of METTL3-depleted mice. Sequencing of m^6^A-modified RNA in vivo and polysome-bound RNA in METTL3-depleted enteroids revealed decreased translation efficiency for methylated transcripts critical to growth factor signaling, including master growth regulator and proto-oncogene, *Kras*. Additional investigation verified a link between METTL3 and *Kras* methylation and protein expression. Our data identify METTL3 as an essential regulator of intestinal TA cell survival via direct support of growth factor signaling, including KRAS expression. By identifying epitranscriptomic regulation as an indispensable process within TA cells, we highlight the importance of an emergent gene-regulatory mechanism in this critical but poorly understood cell type.

## Results

### Intestinal epithelial METTL3 deletion results in complete growth failure and mortality.

**Figure 1:**
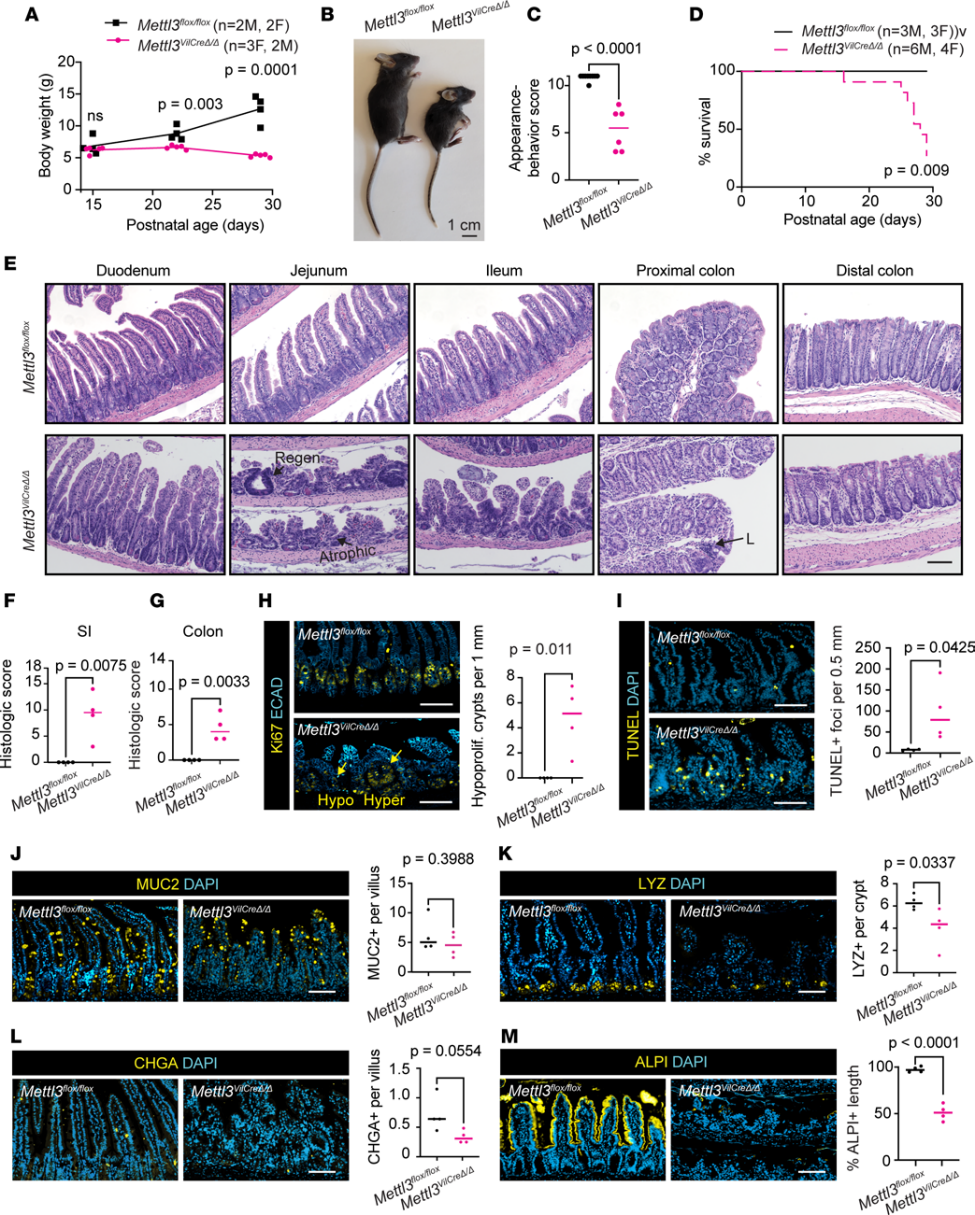
Constitutive METTL3 deletion causes growth retardation and small intestinal epithelial distortion. (**A**) Growth curves from postnatal day 15 to 29. (**B**) Gross appearance at postnatal day 29. (**C**) Composite appearance and behavior score at postnatal day 29. (**D**) Kaplan-Meier survival curves through postnatal day 29; *P* value corresponds to log-rank (Mantel-Cox) test. (**E**) Representative small intestine and colon H&E images. Regenerative and atrophic crypts are highlighted. “L” denotes lymphocytic infiltrate. (**F** and **G**) Composite histological score for small intestine (SI) and colon. (**H**) Representative images of Ki67 in distal small intestine and number of hypoproliferative crypts (< 10 Ki67^+^ cells) per 1 mm distal half small intestine. Hypo- and hyperproliferative crypts are highlighted. (**I**) Representative images and quantification of TUNEL staining. (**J**–**L**) Representative images and quantification of intestinal secretory markers MUC2, LYZ, and CHGA. (**M**) Representative images and quantification of percentage alkaline phosphatase–positive (ALPI) villus length. Each plotted point corresponds to 1 mouse and depicts the mean of 3 representative sections imaged per mouse with bar at median value. Unless otherwise noted, *P* value represents unpaired parametric Student’s *t* test. Immunofluorescence staining and quantification performed in distal half small intestine. All scale bars 100 μm. ECAD, epithelial cadherin; MUC2, mucin 2; LYZ, lysozyme; CHGA, chromogranin A.

**Figure 2:**
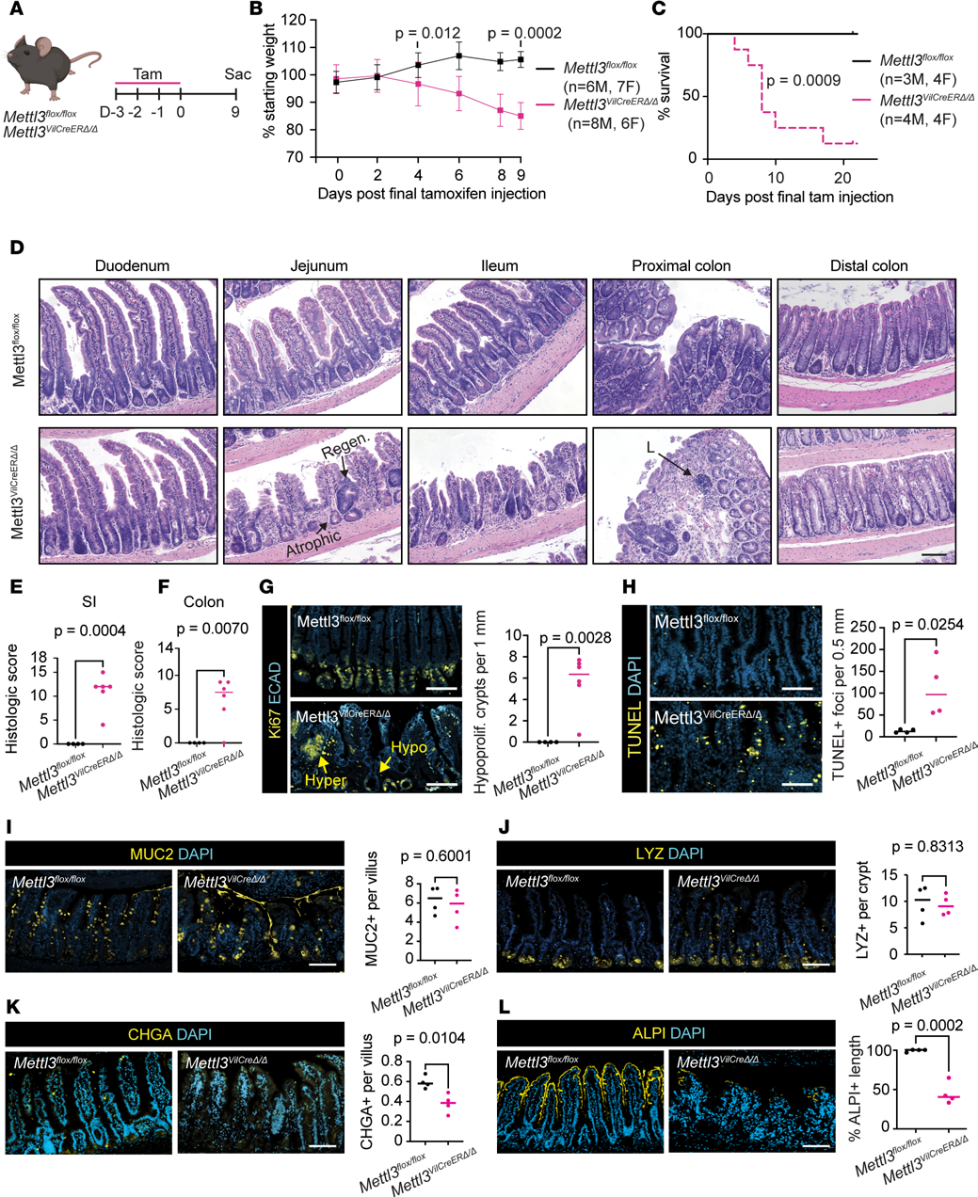
Inducible METTL3 deletion causes mortality and small intestinal epithelial disruption in adult mice. (**A**) Experimental schematic depicting sacrifice 9 days after final tamoxifen injection. (**B**) Weight curves through 9 days after tamoxifen injection, mean ± SD. (**C**) Kaplan-Meier survival curves through 22 days after final tamoxifen injection; *P* value corresponds to log-rank (Mantel-Cox) test. (**D**) Representative small intestine and colon H&E images. Regenerative and atrophic crypts are highlighted. “L” indicates lymphocytic infiltrate. (**E** and **F**) Composite histological score for small intestine (SI) and colon. (**G**) Representative images of Ki67 and quantification of hypoproliferative (< 10 Ki67^+^ cells) crypts per 1 mm intestine. Hypo- and hyperproliferative crypts are highlighted. (**H**) Representative images and quantification of TUNEL staining. (**I**–**K**) Representative images and quantification of intestinal secretory markers MUC2, LYZ, and CHGA. (**L**) Representative images and quantification of percentage alkaline phosphatase–positive villus length. Unless otherwise noted, each data point corresponds to 1 mouse and depicts the mean of 3 representative sections imaged per mouse with bar at median value. Unless otherwise noted, *P* value represents unpaired parametric Student’s *t* test. Immunofluorescence staining and quantification performed in distal half small intestine. All scale bars 100 μm.

To determine the role of METTL3 in intestinal epithelial development and homeostasis, we paired *Mettl3^fl/fl^* mice with the pan-intestinal-epithelial *Villin-Cre* (*Mettl3*^VilCreΔ*/*Δ^) or its tamoxifen-inducible counterpart, *Villin-CreERT2* (inducible *Mettl3*^VilCreERΔ*/*Δ^). First, we examined *Mettl3*^VilCreΔ*/*Δ^ mice, which had constitutive Cre activation in the small intestinal and colonic epithelium beginning at embryonic day 12.5 (26). These mice were born with Mendelian distribution (χ^2^ *n* = 79, *P* > 0.74) and appeared grossly normal at postnatal day 14, as previously described (27). However, from postnatal day 21 to 28, *Mettl3*^VilCreΔ*/*Δ^ mice lost approximately 20% starting body weight while controls gained approximately 40% (Figure 1A). Body condition and weight loss in *Mettl3*^VilCreΔ*/*Δ^ mice required euthanasia of 70% of mice between postnatal day 16–29 (Figure 1, B–D). To determine whether this phenotype was development specific, we next examined inducible *Mettl3*^VilCreERΔ*/*Δ^ mice injected with tamoxifen at 8 weeks of age (Figure 2A). After their final tamoxifen injection, inducible *Mettl3*^VilCreERΔ*/*Δ^ mice exhibited an average daily weight loss of approximately 2.5% (Figure 2B). Within 10 days, almost all mice experienced critical (>20%) weight loss requiring euthanasia (Figure 2, B and C). These data demonstrate a requirement of METTL3 for growth and survival during the postnatal period and adulthood.

### METTL3 deletion induces small intestinal crypt and villus atrophy.

We next examined the intestinal pathology of *Mettl3*^VilCreΔ*/*Δ^ and inducible *Mettl3*^VilCreERΔ*/*Δ^ mice to determine the cause of their severe growth failure and mortality. We assessed *Mettl3*^VilCreΔ*/*Δ^ mice at postnatal day 29 and inducible *Mettl3*^VilCreERΔ*/*Δ^ mice at 9 days after tamoxifen injection, since at this time point both cohorts exhibited daily weight loss and more than 50% of mice met the humane endpoints for euthanasia (see Methods). Western blot, in situ staining, and m^6^A dot blot verified depletion of METTL3 and m^6^A in the intestinal epithelium (Supplemental Figure 1, A–C, and Supplemental Figure 2, A and B; supplemental material available online with this article; https://doi.org/10.1172/jci.insight.171657DS1). We also observed commensurate depletion of METTL14 in METTL3-knockout tissues, which is consistent with previous reports demonstrating stabilization of METTL14 by METTL3 (Supplemental Figure 1A and Supplemental Figure 2A) (28, 29). Using a composite histopathological score in both METTL3-knockout models, we noted histological changes throughout the small intestine and colon with only the distal colon being relatively spared (Figure 1, E–G, and Figure 2, D–F). Defects were most severe in the distal small intestine, where we observed widespread crypt atrophy alongside villus shortening. After METTL3 knockout, villi in the jejunum averaged approximately 30% of their normal length (Supplemental Figure 1D and Supplemental Figure 2C). Many distal small intestinal crypts also degenerated after METTL3 deletion, although we noted occasional hypertrophic regenerative crypts (Figure 1E and Figure 2D). Since METTL3 and METTL14 knockouts are generally considered equivalent, our findings of small intestinal destruction with distal colonic sparing were striking and unexpected given previous studies indicating METTL14 deletion spares the small intestine but induces severe distal colonic defects (23, 24). These data demonstrate that intestinal epithelial METTL3 is required for both the postnatal development and adult maintenance of full-length crypt and villus structures, particularly in the distal small intestine.

### METTL3 is required for intestinal epithelial proliferation and survival.

To determine the origins of crypt and villus atrophy, we next evaluated proliferation and apoptosis in *Mettl3*^VilCreΔ*/*Δ^ and inducible *Mettl3*^VilCreERΔ*/*Δ^ mice. Since histological changes were most severe in the distal small intestine, we focused on Ki67 and TUNEL staining in this tissue. In METTL3-knockout epithelium, atrophied crypts previously observed by H&E exhibited drastically reduced Ki67 staining. Where control crypts had an average of approximately 30 Ki67^+^ cells/crypt, atrophic METTL3-depleted crypts often exhibited fewer than 10 Ki67^+^ cells (Figure 1H and Figure 2G). We also observed some hyperproliferative crypts in both deletion models (>45 Ki67^+^ cells), though in inducible *Mettl3*^VilCreERΔ*/*Δ^ mice, these were frequently METTL3^+^ by immunofluorescence (Supplemental Figure 2D), suggesting small areas of incomplete genetic deletion. Thus, we elected not to quantify these hyperproliferative crypts. We also identified a >10-fold increase in the mean number of TUNEL-positive cells in both *Mettl3*^VilCreΔ*/*Δ^ and inducible *Mettl3*^VilCreERΔ*/*Δ^ mice, demonstrating extensive cell death throughout the villus and crypt (Figure 1I and Figure 2H). These data suggest that both disrupted proliferation and cell survival contributed to epithelial defects in *Mettl3*^VilCreΔ*/*Δ^ and inducible *Mettl3*^VilCreERΔ*/*Δ^ mice.

### METTL3 is required for absorptive enterocyte maturation.

To evaluate etiologies of weight loss and epithelial distortion in METTL3-knockout mice, we examined the distribution of differentiated epithelial cells. The differentiated intestinal epithelium comprises secretory and absorptive lineages, which both play essential roles in intestinal homeostasis. Secretory cell depletion can promote epithelial defects due to important roles in producing mucus, antimicrobial compounds, and stem cell niche factors (30, 31). However, even in areas of severe epithelial distortion, we observed maintenance of MUC2^+^ goblet cells with only minor, inconsistent reductions in LYZ^+^ Paneth cells and CHGA^+^ enteroendocrine cells (Figure 1, J–L, and Figure 2, I–K). Alcian blue staining corroborated these findings by indicating mucus production was also maintained in the small intestine, though we did observe reductions in mucus in the proximal colon (Supplemental Figure 1E and Supplemental Figure 2E). In contrast with modest changes in secretory lineages, we observed a dramatic decrease in expression of absorptive enterocyte marker alkaline phosphatase. Both *Mettl3*^VilCreΔ*/*Δ^ mice and inducible *Mettl3*^VilCreERΔ*/*Δ^ mice exhibited approximately 50% less alkaline phosphatase staining in the distal small intestine, suggesting dramatic loss of absorptive enterocytes (Figure 1M and Figure 2L). This effect was strongest in areas of severe villus shortening, where we observed almost no alkaline phosphatase staining. Taken together, we found general maintenance of secretory cells but a dramatic reduction in mature absorptive enterocytes in *Mettl3*^VilCreΔ*/*Δ^ mice and inducible *Mettl3*^VilCreERΔ*/*Δ^ mice. We propose that the resulting loss of absorptive capacity may underlie wasting in these METTL3-knockout mice.

### Alternative METTL3 deletion recapitulates small intestinal defects.

We performed the above experiments in inducible *Mettl3*^VilCreERΔ*/*Δ^ mice using *loxP* sites spanning 9 exons of the *Mettl3* locus (Supplemental Figure 3A) (32). This large deletion resulted in some reduction of recombination efficiency in the distal colon and, to a lesser degree, the small intestine (Supplemental Figure 2, B and D). We were also surprised to identify a small intestinal defect after METTL3 deletion, because m^6^A depletion via METTL14 deletion produced no small intestinal phenotype in recent publications (23, 24). To address these concerns, we tested a second METTL3 deletion model with *loxP* sites spanning only exon 4 (*Mettl3*^VilCreERΔ*2/*Δ2^, Supplemental Figure 3, A and B) (33). Inducible *Mettl3*^VilCreERΔ*2/*Δ2^ mice demonstrated efficient deletion of METTL3 in all targeted tissues (Supplemental Figure 3C). In this additional model, we verified rapid weight loss and mortality, hypo- and hyperplastic crypts, preservation of secretory goblet and Paneth cells, and absent distal colonic defects (Supplemental Figure 3, D–G). These data further support the conclusion that METTL3 is essential for small intestinal homeostasis.

### Intestinal defects with METTL3 deletion are independent of intestinal microbiota.

**Figure 3:**
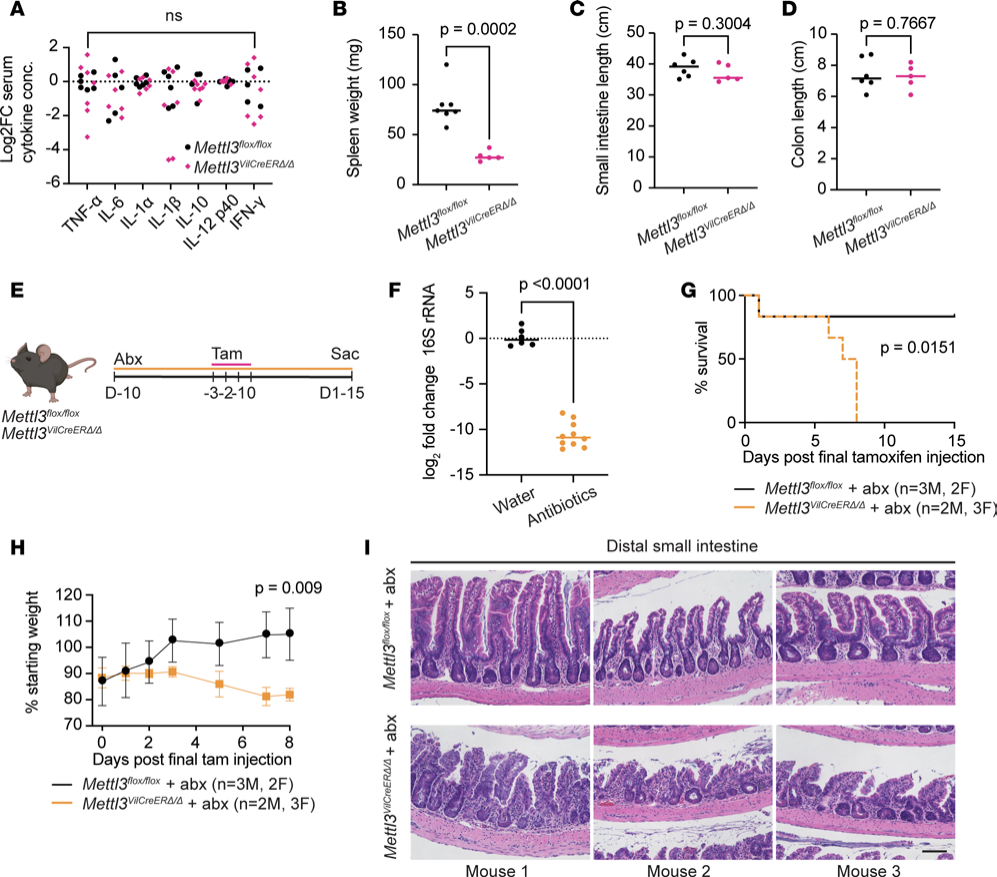
Intestinal distortion and mortality after METTL3 deletion are microbiota independent. (**A**) Log_2_ fold-change in serum cytokines in mice 9 days after final tamoxifen injection; ns indicates *P* > 0.05 for the KO versus control comparison for each individual cytokine. Statistical outliers were removed in GraphPad Prism v9.3 using ROUT method (Q = 1%). (**B**) Spleen weight 9 days after final tamoxifen injection. (**C** and **D**) Small intestine and colon length 9 days after final tamoxifen injection. (**E**) Experimental schematic depicting timing of antibiotic (abx) treatment, tamoxifen injection, and sacrifice. (**F**) Log_2_ fold-change in 16S rRNA amplified from fecal bacterial DNA on final day of tamoxifen injection in antibiotic-treated or water vehicle mice. (**G**) Kaplan-Meier survival curves through 15 days after final tamoxifen injection in antibiotic-treated mice; *P* value corresponds to log-rank (Mantel-Cox) test. (**H**) Weight change after final tamoxifen injection in antibiotic-treated mice, presented as mean ± SD. *P* value represents unpaired parametric Student’s *t* test for values at 8 days after final tamoxifen injection. (**I**) Representative H&E images from matched sections of distal small intestine in *n* = 3 antibiotic-treated mice per genotype. Unless otherwise noted, each data point represents a single mouse with bar at median value, and *P* denotes value of unpaired parametric Student’s *t* test. Scale bar 100 μm.

Microbial translocation and inflammatory activation are common causes of morbidity and mortality in mice with intestinal epithelial defects. Furthermore, we observed modest increases in the number of inflammatory immune cells in the intestinal mucosa of METTL3-depleted mice (Figure 1G and Figure 2F). We therefore examined sera of inducible *Mettl3*^VilCreERΔ*/*Δ^ mice for elevated inflammatory cytokines 9 days after final tamoxifen injection, when mice appeared the most grossly ill as described above. Intriguingly, there was no significant difference in serum cytokine markers TNF-α, IL-6, IL-1α, IL-1β, IL-10, IL-12, or IFN-γ in *Mettl3*^VilCreERΔ*/*Δ^ mice compared to controls (Figure 3A). Additionally, spleen size — often positively correlated with degree of whole-body inflammation — was reduced (Figure 3B), and small intestine and colon lengths — often reduced in inflammatory conditions — were unchanged at this same time point (Figure 3, C and D). Taken together, these data suggested a noninflammatory etiology to morbidity and mortality in *Mettl3*^VilCreERΔ*/*Δ^ mice.

Despite no overt inflammatory pathology, the contribution of facility-specific microbiota is a common concern in the evaluation of intestinal phenotypes. We also questioned whether the microbiota contributed to the surprising difference in small intestinal phenotypes between METTL3 and previously described METTL14-knockout intestinal epithelium (23, 24). Therefore, we depleted the microbiota in *Mettl3*^VilCreERΔ*/*Δ^ mice by adding an antibiotic cocktail to their drinking water beginning 1 week before tamoxifen injection (Figure 3E). Quantitative PCR (qPCR) of genomic 16S rRNA verified approximately 1,000-fold depletion of luminal bacteria (Figure 3F). There was no change in weight loss or mortality in microbiota-depleted *Mettl3*^VilCreERΔ*/*Δ^ mice compared to those on normal drinking water (Figure 3, G and H). Histological abnormalities also persisted in microbiota-depleted *Mettl3*^VilCreERΔ*/*Δ^ mice (Figure 3I). Taken together, these data suggest minimal contribution of inflammation or microbiota to the gross and histological defects seen in *Mettl3*^VilCreERΔ*/*Δ^ mice. Rather, they further support a model in which loss of absorptive enterocytes diminishes digestive capacity, leading to weight loss and mortality in these mice.

### METTL3 deletion immediately triggers TA cell death.

**Figure 4:**
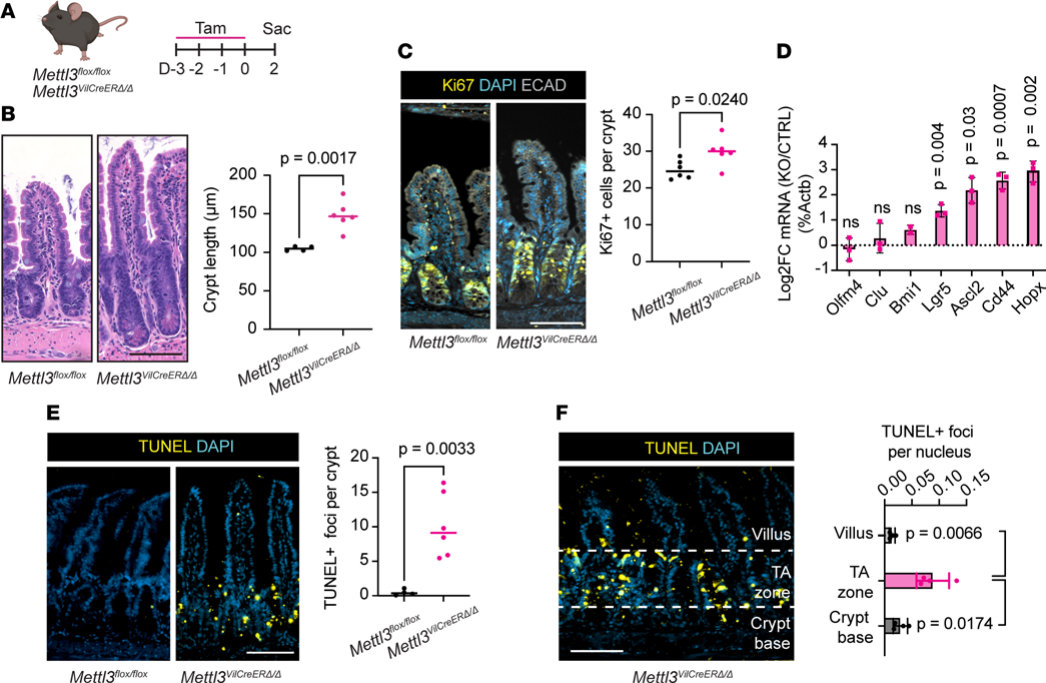
METTL3 deletion rapidly induces death of transit-amplifying cells. (**A**) Experimental schematic depicting early sacrifice 2 days after tamoxifen injection. (**B**) Representative images and quantification of crypt height. (**C**) Representative images and quantification of Ki67 staining. (**D**) Log_2_ fold-change in qPCR quantification of indicated genes in *Mettl3*^VilCreERΔ*/*Δ^ distal small intestinal crypts relative to the mean of *Mettl3^fl/fl^* controls and normalized to *Actb*. Data presented as mean ± SD. (**E**) Representative images and quantification of TUNEL staining. (**F**) Representative image and quantification of distribution of TUNEL staining in villus, transit-amplifying (TA) zone, and crypt base in *Mettl3*^VilCreERΔ*/*Δ^ mice. Data presented as mean ± SD. Each plotted data point corresponds to 1 mouse. For IF, each data point depicts the mean of 3 representative sections imaged per mouse with bar at median value. *P* value represents unpaired parametric Student’s *t* test. All data from distal small intestine of mice 2 days after final tamoxifen injection. All scale bars 100 μm.

Inducible *Mettl3*^VilCreERΔ*/*Δ^ mice displayed a complex set of histological phenotypes, including adjacent hypertrophic and atrophic crypts and scattered villus and crypt cell death. Some of these changes may be reactive, secondary changes rather than immediately downstream of METTL3 depletion. We therefore examined inducible *Mettl3*^VilCreERΔ*/*Δ^ mice 2 days after the final tamoxifen injection — the earliest time point at which we verified METTL3 deletion by immunoblot and in situ staining (Figure 4A and Supplemental Figure 2, A and B). At this early deletion time point, *Mettl3*^VilCreERΔ*/*Δ^ mice demonstrated an approximately 30% increase in crypt height in the small intestine (Figure 4B). This change was associated with increased numbers of Ki67^+^ cells (Figure 4C) and increased expression of transcripts associated with stem cells and stem progenitors, including key stem cell marker *Lgr5* (Figure 4D). In contrast, we observed profound TA cell death in inducible *Mettl3*^VilCreERΔ*/*Δ^ mice at this same early time point. The mean number of TUNEL^+^ foci per crypt was elevated more than 20-fold in *Mettl3*^VilCreERΔ*/*Δ^ crypts compared with controls (Figure 4E). Strikingly, almost all TUNEL^+^ foci in *Mettl3*^VilCreERΔ*/*Δ^ small intestine were in the TA zone, located between the crypt base and the crypt-villus junction. We quantified TUNEL^+^ staining in the crypt base, TA zone, and villus epithelium and found 3-fold higher cell death rates in the TA zone compared with the crypt base (Figure 4F). These data suggest that the initial defect in inducible *Mettl3*^VilCreERΔ*/*Δ^ mice is widespread TA cell death.

### METTL3 deletion causes growth arrest and death in intestinal enteroids.

**Figure 5:**
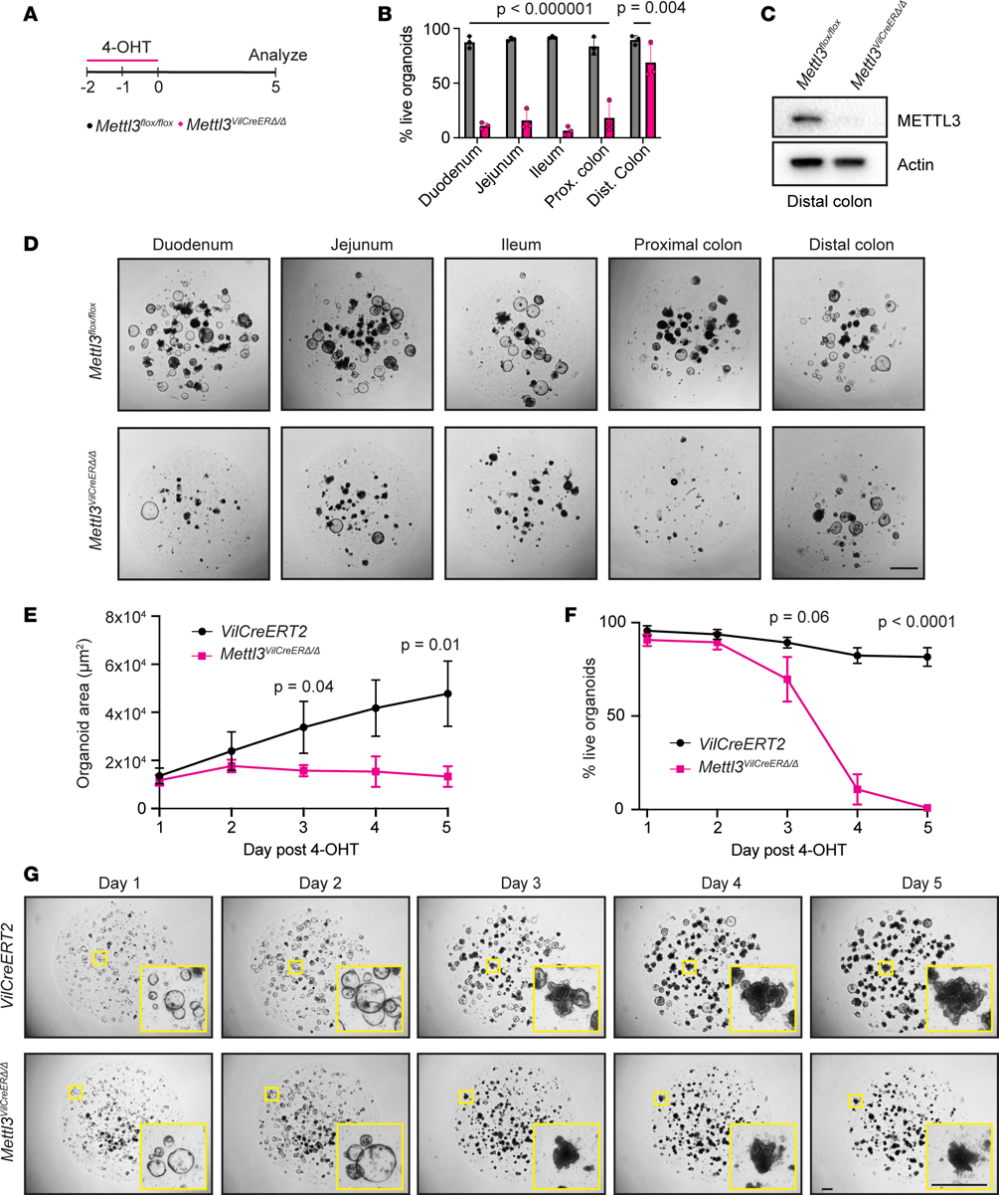
METTL3 deletion triggers growth arrest and death in intestinal epithelial enteroids and colonoids. (**A**) Intestinal epithelial enteroids or colonoids were treated with 1 μm 4-OHT 48 and 24 hours before beginning of time course and analyzed at days 1 through 5. (**B**) Percentage live enteroids or colonoids from indicated tissue regions at 5 days after 4-OHT treatment. Each point represents *n* = 9 technical replicates across *n* = 3 passage separated biological replicates per genotype. Data presented as median ± SD. *P* value represents unpaired parametric Student’s *t* test. (**C**) Western blot for METTL3 in surviving distal colonoids 6 days after 4-OHT treatment. (**D**) Representative images at 5 days after 4-OHT treatment corresponding to quantification in **B**. Scale bar 500 μm. (**E** and **F**) ImageJ (NIH) quantification of average enteroid 2D area and percentage live enteroids in each of the 5 days after 4-OHT treatment of *Villin-CreERT2* (VilCreERT2) and *Mettl3*^VilCreERΔ*/*Δ^ ileal enteroids. Each point represents *n* = 9 technical replicates across *n* = 3 passage-separated biological replicates per genotype. Data presented as mean ± SD. *P* value represents unpaired parametric Student’s *t* test at day 3 and day 5. (**G**) Representative images of ileal enteroids in the 5 days after 4-OHT treatment. Scale bar 200 μm.

We next wanted to establish an epithelial specific model for examining the kinetics and mechanism of cell death in *Mettl3*^VilCreERΔ*/*Δ^ mice. We therefore generated 3D organoids from small intestinal crypts (enteroids) and colonic crypts (colonoids) of *Mettl3*^VilCreERΔ*/*Δ^ and *Mettl3^fl/fl^* mice and treated with 4-hydroxytamoxifen (4-OHT) in vitro to induce METTL3 deletion (Figure 5A) (34). We first monitored gross cell death in enteroids and colonoids. We defined dead organoids as those with a completely opaque appearance, leaking of dead cell debris, and absent growth over the subsequent 24 hours. Nearly all *Mettl3*^VilCreERΔ*/*Δ^ enteroids and proximal colonoids died within 5 days after 4-OHT withdrawal. Mirroring our results in vivo, only distal colonoids from *Mettl3*^VilCreERΔ*/*Δ^ mice survived 4-OHT treatment, where immunoblotting verified deletion of METTL3 (Figure 5, B–D). To map the chronology of enteroid growth and survival after METTL3 deletion, we examined ileal enteroids, which had the greatest rate of death. We measured ileal enteroid size and death daily after 4-OHT treatment of *Mettl3*^VilCreERΔ*/*Δ^ and control *Villin-CreERT2* enteroids. Enteroids appeared grossly normal in the first 2 days after 4-OHT withdrawal. At day 3, *Mettl3*^VilCreERΔ*/*Δ^ enteroids exhibited growth arrest concurrent with increased death, ultimately resulting in complete death by 5 days after 4-OHT (Figure 5, E–G). In summary, *Mettl3*^VilCreERΔ*/*Δ^ enteroids and colonoids recapitulated tissue region–specific cell death phenotypes observed in vivo. These data verified that cell death phenotypes after METTL3 deletion were epithelial cell autonomous; they also established enteroids as an effective model of in vivo pathologies for further mechanistic investigation.

### Catalytic inactive METTL3 does not rescue death of METTL3-depleted enteroids.

Given that METTL3 deletion led to small intestinal epithelial death in vivo and in vitro, but depletion of the essential methyltransferase cofactor METTL14 has been previously reported to have no effect on small intestinal homeostasis (23, 24), we hypothesized that METTL3 might support intestinal epithelial survival through a noncatalytic mechanism. To test this, we reintroduced a noncatalytic METTL3 (DPPW^395-398^ to APPA^395-398^) (35) to *Mettl3*^VilCreERΔ*/*Δ^ ileal enteroids using a lentiviral construct (METTL3^Δcat^). Western and dot blots verified rescue of METTL3 expression, but not m^6^A, in *Mettl3*^VilCreERΔ*/*Δ^ + METTL3^Δcat^ enteroids (Supplemental Figure 4, A and B). However, *Mettl3*^VilCreERΔ*/*Δ^ + METTL3^Δcat^ enteroids still died by day 5 after 4-OHT, similar to negative controls (Supplemental Figure 4, C and D). Conversely, an identical lentiviral construct expressing wild-type METTL3 (METTL3^wt^) restored m^6^A levels and completely rescued death of *Mettl3*^VilCreERΔ*/*Δ^ enteroids (Supplemental Figure 4, A–D). Finally, we considered previous reports indicating that METTL3 may act independently of METTL14 as a noncatalytic cytoplasmic regulator of protein translation (36–38). However, we observed exclusively nuclear staining of endogenous METTL3 in ileal enteroids, likely precluding this possibility (Supplemental Figure 4E). Together, these data suggest that small intestinal epithelium requires the nuclear, catalytic activity of METTL3 for homeostatic function.

### METTL3 deletion triggers global downregulation of translational efficiency.

**Figure 6:**
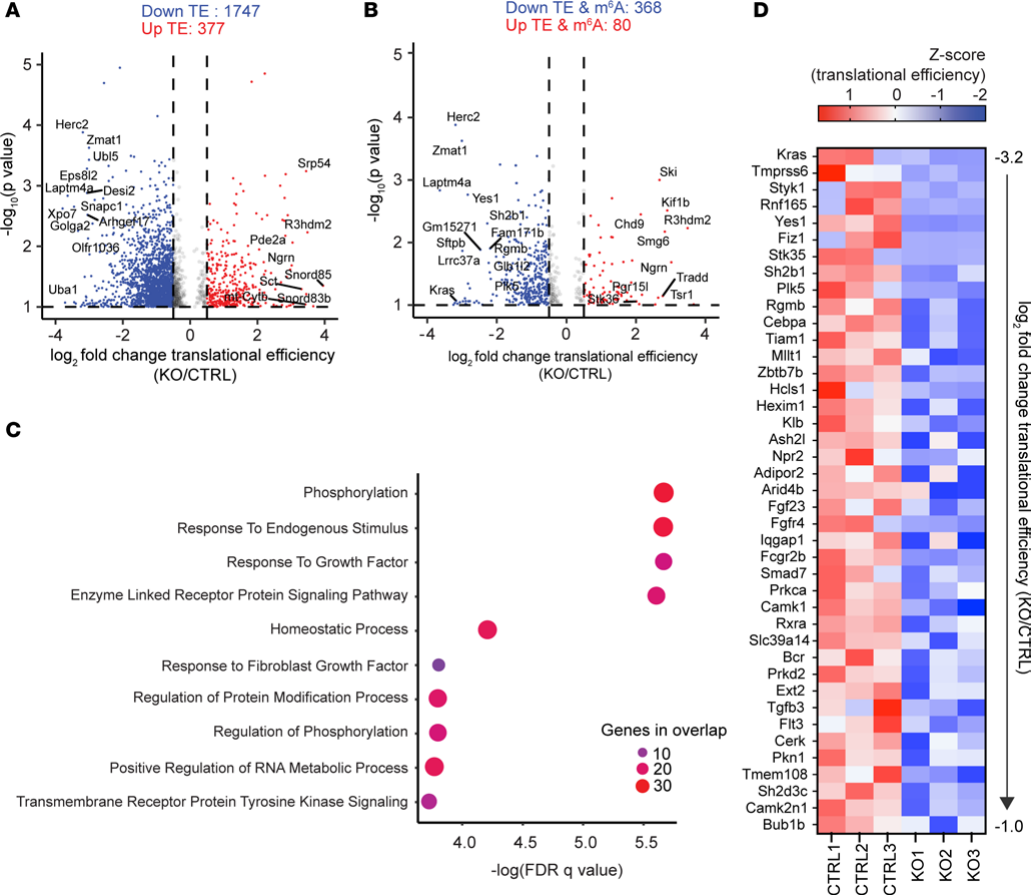
METTL3 deletion leads to a global decrease in mRNA TE with impacts on growth factor signaling. (**A**) Volcano plot of all transcripts with log_2_ fold-change in TE > 0.5 or < –0.5 and –log_10_ *P* > 1. Red marks all transcripts with increased TE, and blue marks all transcripts with decreased TE. (**B**) Volcano plot of all transcripts displayed in **A**, now filtered for transcripts containing at least 1 m^6^A peak. (**C**) Pathway enrichment analysis comparing transcripts with downregulated TE (log_2_FC < –1) and at least 1 m^6^A peak against Gene Ontology Biological Process (GOBP) gene sets. Circle color and size both scale with number of genes overlapping between the tested gene set and the GOBP gene set. (**D**) Heatmap depicting *z* scores for TE. Genes presented are all 42 unique genes from the 4 most significantly enriched pathways in **C**. Genes are presented in order of greatest decrease in mean TE to smallest decrease. All data from RNA-Seq and polysome-Seq in *n* = 3 *Mettl3^fl/fl^* (CTRL) and *n* = 3 *Mettl3*^VilCreERΔ*/*Δ^ (KO) ileal enteroids 72 hours after initiation of 4-OHT treatment.

Previous studies indicate that m^6^A induces global changes in mRNA abundance and translational efficiency (TE) (13, 39, 40). To identify METTL3 targets that may mediate phenotypes observed in vivo and in vitro, we sequenced total RNA and polysome-bound RNA to assay global changes in RNA abundance and translation after METTL3 deletion (Supplemental Data 1 and 2). Quality control analysis of polysome-bound RNA revealed read depth, read length, and interreplicate reproducibility consistent with field standards for polysome profiling (Supplemental Figure 5, A–D). To accurately estimate translation, we compared the ratio of polysome-bound RNA to total mRNA for each transcript to generate the TE of each transcript (11, 41). Since METTL3 modifies thousands of transcripts with pleiotropic effects, we elected to analyze *Mettl3*^VilCreERΔ*/*Δ^ ileal enteroids 72 hours after initiating 4-OHT treatment to detect the changes most proximal to METTL3 deletion but prior to widespread cell death. Comparing the approximately 25,000 transcripts detected in both data sets, METTL3 deletion affected more transcripts at the level of RNA translation (2,124 transcripts) than RNA abundance (1,231 transcripts) (Figure 6A and Supplemental Figure 6A). Averaging all 2,124 transcripts with significant changes in TE, the predominant effect of METTL3 deletion was reduced TE, with 1,747 transcripts exhibiting reduced TE and 377 with increased TE. Taken together, there was a mean decrease in TE of 39% across all differentially translated transcripts in *Mettl3*^VilCreERΔ*/*Δ^ enteroids compared with controls (Figure 6A). These data suggest that METTL3 broadly supports translation in the small intestinal epithelium.

### METTL3 deletion downregulates translation of methylated transcripts regulating growth factor signaling.

We next performed m^6^A immunoprecipitation and sequencing (m^6^A-seq) in wild-type mouse crypts in vivo to define putative direct targets of METTL3 (16, 17, 42). m^6^A-seq yielded 13,763 m^6^A peaks within 7,882 unique transcripts (Supplemental Data 3). Peaks were distributed across the coding sequence and 3′ untranslated region (UTR), with the highest accumulation at the stop codon, consistent with previous reports (Supplemental Figure 7A) (16, 17). We also saw expected patterns of m^6^A enrichment in positive and negative control transcripts (Supplemental Figure 7B). We superimposed m^6^A-seq data onto TE data and found that of the 1,747 transcripts with decreased TE after METTL3 deletion, 368 transcripts contained at least 1 m^6^A peak. These 368 m^6^A-modified transcripts had a median TE decrease of 50% after METTL3 deletion (Figure 6B). By comparison, only 165 m^6^A-modified transcripts had *any* change in RNA abundance after METTL3 deletion, and the magnitude of these changes was approximately half that seen in the TE analysis (Supplemental Figure 6, B and C). Because we observed more changes in RNA translation than abundance after METTL3 deletion, we chose to focus our remaining analysis on changes in TE. We next used Gene Ontology Biological Process (GOBP) gene sets to conduct pathway enrichment analysis on these 368 methylated transcripts with downregulated TE after METTL3 deletion. We observed the most significant enrichment in transcripts associated with growth factor signaling cascades (Figure 6C). We observed broadly reduced TE in growth factor–associated transcripts (Figure 6D). Notably, the largest magnitude reduction in TE was for *Kras*, an essential intestinal proto-oncogene that promotes intestinal epithelial proliferation and survival (43). Taken together, sequencing of polysome-bound and m^6^A-modified mRNA indicates that METTL3 deletion downregulates translation of methylated transcripts supporting growth factor signaling, including *Kras*.

### METTL3 deletion reduces Kras methylation and protein levels and induces cellular senescence.

**Figure 7:**
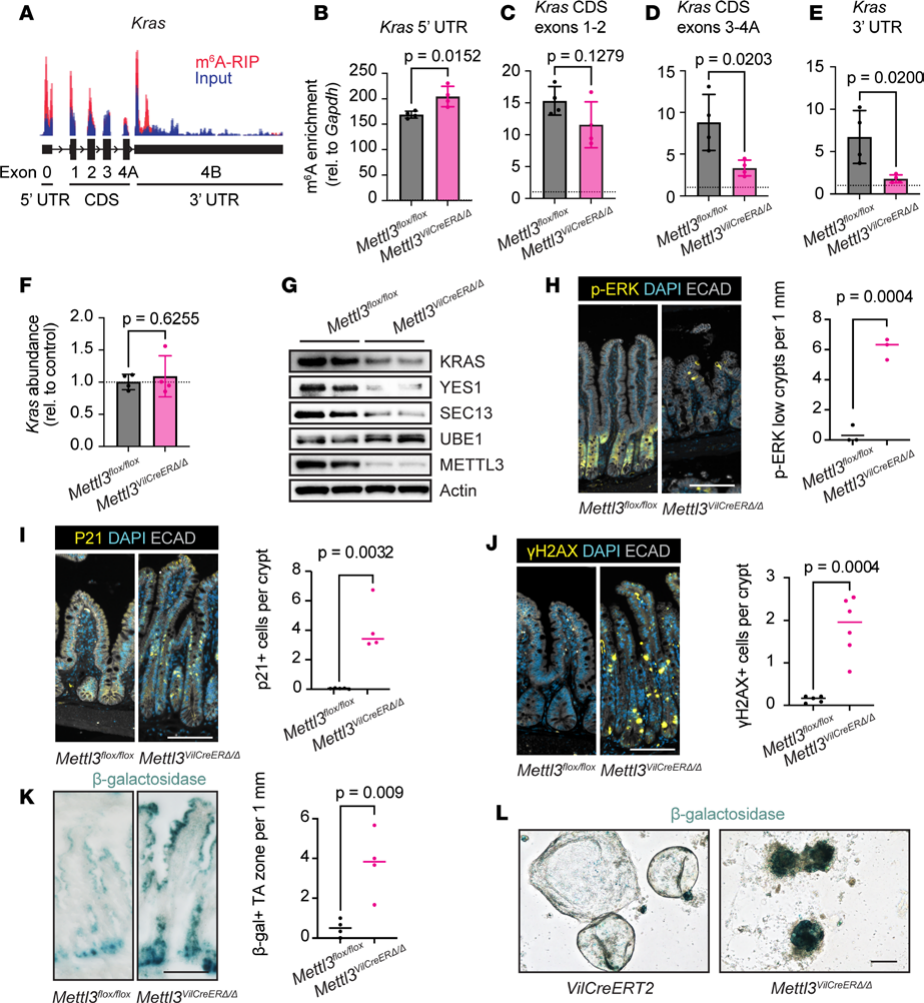
METTL3 deletion downregulates KRAS and induces cellular senescence. (**A**) Integrated Genomics Viewer depiction of read density for m^6^A-RIP (red) and input RNA (blue) for the *Kras* transcript as determined by m^6^A-seq in distal small intestinal crypts of *n* = 3 wild-type mice. CDS, coding sequence; RIP, RNA immunoprecipitation. (**B**–**E**) m^6^A enrichment determined by m^6^A-RIP-qPCR with primers targeting *Kras* 5′ UTR, CDS exons 1–2, CDS exons 3–4A, and 3′ UTR in crypt-enriched lysates from *Mettl3^fl/fl^* and *Mettl3*^VilCreERΔ*/*Δ^ mice 3 days posttamoxifen. Data presented as mean ± SD. Dotted line at m^6^A enrichment = 1. (**F**) qPCR for *Kras* transcript in crypt-enriched lysates from *Mettl3^fl/fl^* and *Mettl3*^VilCreERΔ*/*Δ^ mice 3 days posttamoxifen. Data normalized to *Actb* and the mean of *Mettl3^fl/fl^* controls. Data presented as mean ± SD. (**G**) Western blot for top targets with downregulated TE in crypts of *Mettl3^fl/fl^* and *Mettl3*^VilCreERΔ*/*Δ^ mice 2 days after final tamoxifen injection (*n* = 2 mice per genotype). (**H**) Representative images and quantification of p-ERK staining in atrophic small intestinal crypts in *Mettl3*^VilCreERΔ*/*Δ^ mice and region-matched *Mettl3^fl/fl^* controls 9 days after final tamoxifen injection. “p-ERK low” crypts contain < 5 p-ERK^+^ cells. (**I**–**K**) Representative images and quantification of p21, γH2AX, and β-galactosidase staining in distal half small intestine of *Mettl3^fl/fl^* and *Mettl3*^VilCreERΔ*/*Δ^ mice 2 days after final tamoxifen injection. (**L**) β-Galactosidase staining in control *VilCreERT2* and *Mettl3*^VilCreERΔ*/*Δ^ enteroids 3 days after 4-OHT. For all plots, each data point represents a single mouse, and *P* denotes value of unpaired parametric Student’s *t* test. Unless otherwise noted, immunofluorescence data are from areas of most severe histological distortion in distal small intestine of mice 2 days after final tamoxifen injection. For immunofluorescence, each data point is the mean of 3 representative sections imaged per mouse with bars at median value. Scale bar 100 μm.

We further explored the putative relationship between METTL3 and KRAS expression by examining m^6^A modification of the *Kras* transcript. Our m^6^A-seq data indicated enriched m^6^A density across the *Kras* gene body, including both UTRs (Figure 7A). However, to our knowledge, previous literature had never identified m^6^A methylation of *Kras*. To verify m^6^A-seq peaks on the *Kras* transcript and determine their dependence on METTL3 expression, we performed m^6^A-RIP-qPCR in crypt-enriched lysates from *Mettl3^fl/fl^* and *Mettl3*^VilCreERΔ*/*Δ^ mice 3 days after final tamoxifen injection. We found substantial enrichment of m^6^A in all *Kras* mRNA regions using 4 unique qPCR probes targeting the *Kras* 5′ UTR, CDS exons 1–2, CDS exons 3–4A, and 3′ UTR (Figure 7, B–E). However, close examination of our data suggested that the true m^6^A peak occupied the 3′ UTR only. Of the 4 regions sampled, m^6^A enrichment appeared dependent on METTL3 expression only in CDS exons 3–4A and the 3′ UTR (Figure 7, D and E). Of these 2 regions, only the 3′ UTR demonstrated clear m^6^A enrichment in the m^6^A-seq (Figure 7A). The low resolution of the m^6^A-RIP-qPCR (~200–300 nt) could explain why we observed m^6^A enrichment in CDS exons 3–4A. The m^6^A enrichment in the 5′ UTR and neighboring CDS exons 1 to 2 (Figure 7, B and C) likely represent N^6^,2′-O-dimethyladenosine (m^6^Am), a terminal modification added to the 5′ mRNA cap independent of METTL3; m^6^Am is an established off target of m^6^A antibodies (44). These data support our original finding that METTL3 methylates *Kras* in the 3′ UTR.

We next examined how methylation of *Kras* affects its expression in vivo. Although METTL3 deletion did not significantly affect *Kras* transcript abundance via qPCR, we observed a decrease in KRAS protein in crypt-enriched lysates from *Mettl3*^VilCreERΔ*/*Δ^ mice compared with controls (Figure 7, F and G). To further validate our polysome-sequencing data in vivo, we also examined protein expression levels of other top downregulated genes of interest. We verified decreases in YES1 (src family kinase and proto-oncogene) and SEC13 (member of the nuclear pore complex implicated in mRNA export) but not UBE1 (primary enzyme in conjugation of ubiquitin) (45–47) (Figure 7G). Finally, to support our model of reduced KRAS expression, we examined the distal small intestine of inducible *Mettl3*^VilCreERΔ*/*Δ^ mice for levels of key downstream target phosphorylated ERK (p-ERK). Control crypts averaged between 30 and 40 p-ERK–positive cells, predominantly in the TA zone. By contrast, about 50% of atrophic small intestinal crypts in *Mettl3*^VilCreERΔ*/*Δ^ mice had fewer than 5 p-ERK–positive cells per crypt after deletion of METTL3 (Figure 7H). Taken together, these data suggest that METTL3 promotes KRAS expression and signaling without impacting *Kras* transcript levels, providing further mechanistic support for METTL3 as a posttranscriptional regulator of *Kras* translation.

KRAS is a key mediator of epidermal growth factor (EGF) signaling in epithelial cells (43). Loss of mitogenic signals such as EGF induces cell cycle arrest and senescence in proliferative cells (48). We therefore assessed senescence in the crypts of inducible *Mettl3*^VilCreERΔ*/*Δ^ mice by staining for p21, γ-H2AX, and β-galactosidase 2 days after final tamoxifen injection. *Mettl3*^VilCreERΔ*/*Δ^ crypts demonstrated elevated p21, γ-H2AX, and β-galactosidase compared with controls, particularly in the TA zone (Figure 7, I–K). Dying *Mettl3*^VilCreERΔ*/*Δ^ enteroids also exhibited strong β-galactosidase staining compared with controls (Figure 7L). One potential caveat to these findings is the possibility of toxic combined effects of CreERT2 and tamoxifen in intestinal stem cells within a week of tamoxifen exposure (49). To address this, we evaluated p21 and γ-H2AX in tamoxifen-treated *Villin-CreERT2* control mice at the same time point. Both p21 and γ-H2AX were significantly increased in *Mettl3*^VilCreERΔ*/*Δ^ mice compared with *Villin-CreERT2* controls (Supplemental Figure 8, A and B). Although we propose that KRAS downregulation is not the sole event responsible for the death of METTL3 KO epithelium, we examined whether overexpression of KRAS could rescue death of METTL3-KO enteroids to definitively determine the role of KRAS expression. We introduced vectors expressing either KRAS or constitutively active KRAS^G12D^ to *Mettl3*^VilCreERΔ*/*Δ^ enteroids and induced METTL3 KO. Unsurprisingly, neither KRAS nor constitutively active KRAS^G12D^ could rescue *Mettl3*^VilCreERΔ*/*Δ^ enteroids (Supplemental Figure 9, A–C). Taken together, these data support the hypothesis that loss of proteins supporting growth factor signaling, including but not limited to KRAS, triggers cellular senescence in the crypt TA zone after METTL3 deletion.

## Discussion

**Figure 8:**
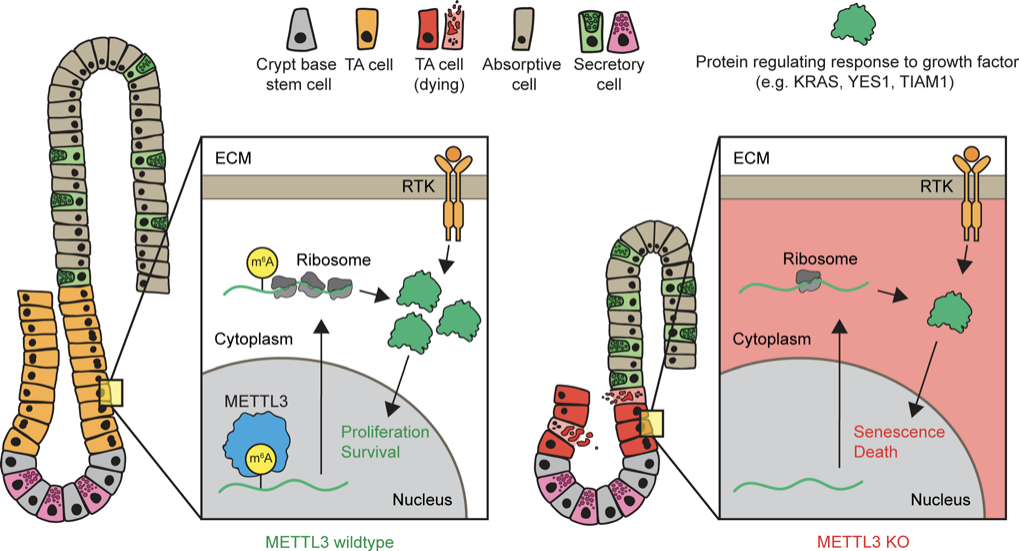
METTL3 maintains growth factor signaling and survival in intestinal transit-amplifying cells. Proposed model. In wild-type mice, METTL3 methylates *Kras* and other transcripts involved in transducing growth factor signaling. Methylation promotes translation of these transcripts, enhancing proliferation and survival in transit-amplifying (TA) cells downstream of external growth factors. In the absence of METTL3, a decreased response to extracellular growth factors in METTL3-knockout TA cells leads to cellular senescence and death. Loss of transit amplification results in reduced crypt and villus size and diminished production of absorptive cells. Green protein represents KRAS and other proteins regulating the intracellular response to growth factors. ECM, extracellular matrix; RTK, receptor tyrosine kinase.

Our collective findings examining METTL3 deletion in the intestinal epithelium in vivo and in vitro support a model in which METTL3 methylates the *Kras* transcript and other transcripts involved in growth factor signal transduction. Methylation promotes translation of these transcripts, maintaining responsiveness to extracellular growth factors and thus maintaining proliferation and survival in the TA cells of the crypt. TA cells make up most of the crypt, and TA cell proliferation sustains the production of the absorptive enterocytes of the villus. Therefore, TA cell death reduces crypt and villus size and diminishes absorptive cell numbers without substantially impacting secretory cells, which are less dependent on transit amplification (4) (Figure 8).

We originally expected that METTL3 deletion would have no overt effect on the small intestinal epithelium because previous reports showed that METTL14 was dispensable in the small intestine (23, 24). Surprisingly, we found that small intestinal homeostasis requires METTL3. However, our study does not directly compare METTL3 and METTL14. Therefore, discrepancies between the present study and previously described METTL14 knockout studies could be due to facility-specific differences (e.g., microbiota). Several factors minimize this possibility. First, 2 independent groups from the United States and China reported colonic but not small intestinal phenotypes with METTL14 knockout, demonstrating persistent phenotypes across mouse facilities. Furthermore, we found small intestinal phenotypes followed METTL3 deletion when testing both constitutive and inducible Cre drivers, 2 *Mettl3*-floxed lines, microbiota-depleted mice, and mouse-derived enteroids. The consistency of small intestinal phenotypes spanning numerous orthogonal modes of METTL3 deletion strongly suggests that METTL3 and METTL14 act independently in the small intestine. This unexpected finding suggests divergent roles for METTL3 and METTL14 in the gut and underscores the value of examining m^6^A writer proteins in their native context.

Despite their critical role in maintaining homeostatic renewal of the intestinal epithelium, TA cell dynamics remain poorly understood. We propose that METTL3 is essential for both differentiation and renewal in the TA zone of the small intestinal crypt. As the site of stem progenitor differentiation, TA cells are critical to the process of lineage commitment in the cell (2). We observed general maintenance of secretory cells adjacent to areas of atrophic crypts in *Mettl3^VilCre^* and *Mettl3*^VilCreERΔ*/*Δ^ mice. In contrast, these histologically affected areas demonstrated little to no absorptive cell staining. Since mature absorptive cells make up most of the villus surface, transit amplification in the crypt is essential for the production of adequate numbers of absorptive progenitors rather than secretory cells (50). Consistent with our data showing decreased enterocyte maturation concurrent with loss of TA cells, a recent study suggested a causal relationship between decreased TA proliferation and an increase in the ratio of secretory to absorptive cells (4). Therefore, we propose that METTL3 promotes the production of absorptive cells by maintaining TA cell proliferation.

Inducible METTL3 deletion was also associated with extensive cell senescence and a more than 20-fold increase in the rate of cell death in the small intestinal crypt, especially in cells of the TA zone. TA cell death occurred only 2 days after deletion of METTL3, exceeded rates of cell death in the crypt base 3-fold, and occurred at a time point when markers of crypt base stem cells (e.g., *Lgr5*) were preserved or even increased. Therefore, we conclude that METTL3 deletion causes an independent defect in TA cells. In a very recent publication, Liu et al. describe loss of LGR5^+^ intestinal epithelial stem cells 3 days after METTL3 deletion; they attribute loss of these stem cells to loss of pro-stemness transcription factors regulated by METTL3 (25). It is possible that defects in crypt base stem cells drive TA cell death rather than an intrinsic defect in TA cells, and we do not deny the possibility that crypt base stem cells require METTL3. However, we note that Liu et al. also observed extensive TUNEL staining in METTL3-depleted TA cells 2 days before they found a reduction in the number of crypt base stem cells, corroborating our findings of primary TA cell death. Furthermore, a recent study posited that TA cells regulate the proliferation of LGR5^+^ stem cells by controlling the secretion of R-spondins (51). Thus, an initial defect in the TA zone could also precipitate the loss of LGR5^+^ crypt base stem cells. We therefore view the work of Liu et al. as both complementary to, and supporting of, our own findings. Our data, with the support of Liu et al., suggest that METTL3 specifically regulates the survival of intestinal TA cells.

Rapid proliferation in the TA zone suggests enhanced dependence on mitogenic factors in these cells. Consistent with this hypothesis, METTL3 deletion downregulated translation of multiple methylated transcripts involved in growth factor signaling, including growth factor receptor *Fgfr4* (52), src family kinase *Yes1* (45), guanine nucleotide exchange factor *Tiam1* (53), and *Kras*. KRAS is a target of particular interest because of its role as an oncogene in the gut (43), where up to 50% of colorectal cancers harbor a KRAS mutation (54). KRAS binds to guanosine 5′-triphosphate (GTP) in response to extracellular signals such as epidermal growth factor receptor activation. Activated KRAS-GTP then upregulates growth factor targets such as PI3K-AKT, RAF-MEK-ERK, and the GLUT1 glucose transporter (43). Although frequently dysregulated in cancer, these pathways are also essential for homeostatic proliferation, differentiation, and survival (55). Our data support a direct relationship between METTL3, *Kras* m^6^A methylation, and KRAS protein levels. We posit that large-scale depletion of growth factor signaling — including but not limited to KRAS — overwhelms cells of the TA zone and leads to cell death with METTL3 deletion.

In summary, we identify METTL3 as an essential posttranscriptional regulator of growth factor signaling and cell survival in TA cells, a critical but understudied cell type. This paves the way for future investigation of therapeutic targets that support TA cell resilience and function, including in the setting of DNA-damaging agents such as chemotherapeutics and irradiation.

## Methods

### Animals.

*Mettl3*^VilCreΔ*/*Δ^ and *Mettl3*^VilCreERΔ*/*Δ^ were generated by crossing *Villin-Cre* mice (JAX 021504) or *Villin-CreERT2* mice (JAX 020282) to previously described *Mettl3^fl/fl^* mice (32) provided by Richard Flavell (Yale, New Haven, Connecticut, USA) via Brian Capell (University of Pennsylvania, Philadelphia, Pennsylvania, USA). *Mettl3*^VilCreERΔ*2/*Δ2^ were generated by crossing *Villin-CreERT2* mice to previously described *Mettl3^flox2/flox2^* mice (33), provided by Federica Accornero (The Ohio State University, Columbus, Ohio, USA). For the m^6^A-seq experiment, wild-type mice were used (JAX 000664). All mice were C57BL/6J strain and both male and female mice were used. Mice were housed in a temperature-controlled room with 12-hour light/12-hour dark cycles and continuous access to food and water.

### Tamoxifen injection, euthanasia criteria, and survival curves.

Mice aged 8 to 9 weeks were injected 4 times with 50 mg/kg tamoxifen at 10 mg/mL in corn oil at 24-hour intervals. Mice were euthanized once they had reached humane endpoints defined by the IACUC protocol, including > 20% body weight loss, hunched posture, emaciated body condition, or nonresponsiveness to stimuli. Survival curves were determined by the number of mice reaching a humane endpoint on each day after tamoxifen injection. To assay inflammatory phenotype, spleen, intestines, and serum cytokines were measured in mice euthanized 9 days after final tamoxifen injection, when all mice displayed daily reductions in weight and reached or approached critical body weight loss.

### Microbial depletion.

*Mettl3^fl/fl^* and *Mettl3*^VilCreERΔ*/*Δ^ mice were moved to new cages at 7 weeks of age and given sterile deionized water supplemented with 0.5 g/L ampicillin (MilliporeSigma A9518), 0.5 g/L neomycin (MP Biomedicals 180610), 0.5 g/L gentamicin (MilliporeSigma G1914-5G), 0.25 g/L vancomycin (VWR 0990), 0.25 g/L metronidazole (Thermo Fisher Scientific 210340050), and 4 g/L Splenda to enhance taste. After 7 days, mice were injected with tamoxifen for 4 days as described above, and stool was collected on the final day of injection. Stool from *Mettl3^fl/fl^* and *Mettl3*^VilCreERΔ*/*Δ^ mice on normal drinking water was used as controls. DNA was extracted from stool using the QIAamp Fast DNA Stool Mini Kit (QIAGEN 51604), and qPCR was performed as described in the PCR Methods section using primer Ba04230899_s1. qPCR quantification was normalized to stool weight.

### Serum cytokine quantification.

Serum supernatant was isolated from the inferior vena cava of euthanized mice, and cytokines measured using the Cytometric Bead Array (BD Biosciences), with the amount of capture beads, detection reagents, and sample volumes scaled down 10-fold from manufacturer’s protocol. Data were collected on an LSRFortessa flow cytometer (BD Biosciences) with FACSDiva v9.0 (BD Biosciences) and analyzed with FlowJo v10 (BD Biosciences). Statistical outliers were removed in GraphPad Prism v9.3 using ROUT method (Q = 1%). Cytokines used were mouse TNF-α (BD Biosciences 558299), mouse IL-6 (BD Biosciences 558301), mouse IL-1α (BD Biosciences 560157), mouse IL-1β (BD Biosciences 560232), mouse IL-10 (BD Biosciences 558300), mouse IL-12/IL-23p40 (BD Biosciences 560151), and mouse IFN-γ (BD Biosciences 558296) with Mouse/Rat Soluble Protein Master Buffer Kit (BD Biosciences 558266).

### Histology and immunofluorescence staining.

Intestines were Swiss-rolled and fixed overnight in 4% paraformaldehyde at 4°C prior to processing and embedding. Sections were blocked with blocking buffer (1% BSA and 10% donkey serum in PBS) for 1 hour, at 25°C, before staining with primary antibodies (Supplemental Table 1) at 1:200 overnight at 4°C followed by washing with PBS and staining with secondary antibodies at 25°C for 25 minutes. Finally, nuclei were counterstained with 1:10,000 DAPI and coverslips mounted with ProLong Gold Antifade Reagent (Thermo Fisher Scientific). Duodenum, jejunum, and ileum were defined as the proximal, middle, and distal thirds of the small intestine. Proximal colon was defined as the proximal 4 cm of the large intestine and distal colon as the distal 4 cm. For alkaline phosphatase staining, we used the Vector Red Substrate Kit, Alkaline Phosphatase (Vector Laboratories SK-5100), on deparaffinized slides as described in the manufacturer’s protocol. TUNEL was detected using the Click-iT Plus TUNEL Assay for In Situ Apoptosis Detection, Alexa Fluor 594 dye (Thermo Fisher Scientific C10618). For the β-galactosidase staining, fresh frozen sections were stained overnight with the Senescence β Galactosidase Staining Kit (Cell Signaling Technology 9860) per the manufacturer’s protocol. All stains were imaged on the Keyence BZ-X100.

### Histopathology and appearance scoring.

Histopathological scoring was performed in a blinded manner by an anatomical pathologist based on previously published criteria with an additional score for villus damage for small intestine (Supplemental Table 2) (56). For each category, the recorded score reflects the most severe finding. For percentage involvement, the recorded score reflects the length of bowel that was involved by any of the scored processes. Scores were summed for each mouse to generate a separate composite score for small intestine and colon. Mouse appearance/behavior was scored according to a previously described rubric (57).

### Intestinal epithelial organoid cultures.

Intestinal sections were splayed open, rinsed in PBS, and rotated at 4°C for 45 minutes in HBSS with 10 mM EDTA and 1 mM N-Acetyl-l-cysteine (MilliporeSigma A9165). Crypts were isolated by scraping with a glass coverslip followed by vortexing and filtering through a 70 μM (small intestine, CELLTREAT 229483) or 100 μM (colon, CELLTREAT 229485) cell strainer. Crypt-enriched suspensions were centrifuged at 500*g*, 25°C, for 5 minutes; washed in PBS; and pelleted again at 500*g*, 25°C, for 5 minutes. Crypts were plated in Matrigel droplets (Corning 354234) and, unless stated otherwise, overlaid with the following “ENR” medium: advanced DMEM/F12 media (Thermo Fisher Scientific 12634028) containing 1× GlutaMAX (Thermo Fisher Scientific 35050061), 10 mM HEPES (Thermo Fisher Scientific 15-630-080), 1× Antibiotic Antimycotic (Thermo Fisher Scientific 15240062), 1× N-2 Supplement (Thermo Fisher Scientific 17502048), 1× B-27 Supplement (Thermo Fisher Scientific 17504044), 5 μM CHIR99021 (Cayman Chemical 13122), 1 mM N-Acetyl-l-cysteine (MilliporeSigma A9165), 50 ng/mL mEGF (PeproTech 315-09), 5% Noggin/R-spondin conditioned medium (generated using protocol from ref. 34), and 10 μM Y-27632 (LC Labs Y-5301). Colonic epithelial cultures were fed 50% WRN CM media (58). Passage-matched enteroids/colonoids were used for all experiments. For induction of CreERT2, enteroids/colonoids were given 1 μM 4-OHT in 100% ethanol at 48 and 24 hours before the start of the time course, then mechanically passaged at day 0.

### Whole-mount staining in enteroids.

Enteroids were dissociated to single cells using Accutase (STEMCELL Technologies 07920), resuspended in ENR supplemented with 10 μM Y-27632, and plated on chamber slides pretreated with 10% Matrigel in Advanced DMEM/F12. The next day, slides were incubated in 4% paraformaldehyde at room temperature for 20 minutes and blocked in 10% goat serum in PBS for 30 minutes at 25°C. We used 1:200 rabbit monoclonal anti-METTL3 (Abcam ab195352) and 1:200 goat polyclonal anti–E-Cadherin (R&D Systems AF748) at 37°C for 30 minutes, followed by 1:600 Alexa Fluor 488 AffiniPure Bovine Anti-goat IgG (Jackson Laboratory 805-545-180) and Cy3 AffiniPure Donkey Anti-Rabbit IgG (Jackson Laboratory 711-165-152) in PBS supplemented with 1:5,000 DAPI. Chambers were detached from the chamber slide and coverslips mounted with ProLong Gold Antifade Reagent. For the β-galactosidase staining, enteroids were grown embedded in Matrigel thinly smeared on standard tissue culture plates and stained overnight with the Senescence β Galactosidase Staining Kit per the manufacturer’s protocol. All stains were imaged on the Keyence BZ-X100.

### qRT-PCR.

RNA was isolated using the Quick-RNA Miniprep Kit (Zymo R1054). RNA was reverse-transcribed using MultiScribe Reverse Transcriptase (Thermo Fisher Scientific 4311235) with random hexamer primers. qPCR was performed using the Applied Biosystems TaqMan Fast Advanced Master Mix (Thermo Fisher Scientific 4444556) with TaqMan Gene Expression Assay (FAM) primers (Supplemental Table 3) on an Applied Biosystems QuantStudio 3.

### Western blotting.

Cells were lysed using RIPA buffer (Cell Signaling Technology 9806S) supplemented with 1:100 protease phosphatase inhibitor (Cell Signaling Technology 5872S), and protein concentration was measured using the Pierce BCA Protein Assay Kit (Thermo Fisher Scientific 23225). Lysates were boiled for 5 minutes with 100 mM DTT and 1× LDS buffer (GenScript M00676) and run on NuPAGE 4% to 12% Bis-Tris gels (Thermo Fisher Scientific NP0335BOX) in 1× MOPS-SDS buffer (BioWorld 10530007-2) or 1× MES-SDS buffer (Thermo Fisher Scientific NP0002, for low MW proteins). Gels were transferred onto PVDF transfer membrane in 2× NuPAGE transfer buffer (Thermo Fisher Scientific NP006) and membranes blocked for 1 hour in 5% milk in TBS-T (TBS, 0.1% Tween-20), then placed in 1:1,000 primary antibody (Supplemental Table 1) overnight in 5% milk in TBS-T at 4°C. Blots were washed in TBS-T and placed in 1:2,000 secondary antibody (Supplemental Table 1) for 1 to 2 hours at 25°C. Blots were then subjected to SuperSignal West Femto Maximum Sensitivity Chemiluminescent Substrate (Thermo Fisher Scientific) and imaged on the Bio-Rad Gel Doc XR.

### m^6^A dot blot.

mRNA was isolated using the Dynabeads mRNA DIRECT Purification Kit (Thermo Fisher Scientific 61011). More than 30 ng of mRNA was heated to 65°C for 2 minutes, placed on ice for more than 5 minutes, pipetted onto a Hybond N+ nitrocellulose membrane, and exposed to 254 nm UV light for 5 minutes. The nitrocellulose membrane was then washed in TBS-T. Blots were then incubated in primary and secondary antibody and developed as described in *Western blotting*. We detected m^6^A using anti-m^6^A primary antibody (Cell Signaling Technology 56593). Total blotted mRNA was stained using 0.04% Methylene Blue (LabChem LC168508) in 0.5 M sodium acetate.

### RNA-Seq and polysome-Seq.

Ileal enteroids were expanded in Matrigel in 50% WRN media until 8 to 10 million cells per replicate was achieved. Passage-separated replicates from the same enteroid line were used. Media were supplemented with 2 μM 4-OHT 72 hours prior to collection. Enteroids were collected by mechanical dissociation, pelleted, and snap-frozen in liquid nitrogen. Frozen enteroid pellets were resuspended in ice-cold lysis buffer containing 20 mM Tris-HCl pH 7.4, 150 mM MgCl_2_, 150 mM NaCl, 100 μg/mL cycloheximide, 1% v/v Triton X-100, 1 mM DTT, 1 U/μL SUPERase·IN RNase inhibitor (Thermo Fisher Scientific), 25 U/mL Turbo DNase 1 (Thermo Fisher Scientific), and 1× EDTA-free protease inhibitor cocktail (Roche). Cells were lysed by trituration through a 26-gauge needle 10 times. Samples were processed and libraries prepared as previously described (41) with the following modifications. First, we performed sucrose cushion to pellet the ribosome-associated mRNAs and proceeded with RNase I digestion (10 U/μL) for 30 μg of RNA. Samples were incubated at room temperature for 45 minutes with gentle agitation, and the digestion was quenched by adding 200 units of SUPERase·IN. Then, we directly extracted the ribosome-protected fragments (RPFs) using the TRIzol reagent and performed gel size selection of the RPFs of 15 to 35 nt in length. Second, we performed rRNA depletion using RiboCop for Human/Mouse/Rat kit (Lexogen). Once the rRNA-depleted RPFs were obtained, they were pooled together and the libraries prepared with unique molecular identifiers for deduplication. The multiplexed library was then sequenced on Illumina HiSeq 4000 with PE150 runs (paired end reading of 150 bases), with a sequencing depth of 60 million raw reads/sample. For each sample, one-tenth (150 μL) of the lysate was saved for RNA-Seq. For RNA-Seq, total RNA was extracted using TRIzol LS reagent (Ambion) and purified using Quick-RNA Microprep kit (Zymo) following the manufacturer’s protocol. Libraries were prepared from total RNA using the Smarter Stranded Total RNA-Seq Kit v2 - Pico Input Mammalian (Takara Bio 634411) and sequenced on a NovaSeq 6000, SP Reagent Kit 1.5 (100 cycles). Raw sequencing data were demultiplexed, adaptors were removed using cutadapt (59), contaminant sequences (rRNA and tRNA) were depleted, and reads were deduplicated (umi_tools dedup). Reads were aligned to all transcripts on the mouse reference chromosomes (Gencode version M31, GRCm39) using Kallisto (60). Analysis of ribosome-bound transcripts revealed many transcripts smaller than the expected 29 nt ribosome footprint size (Supplemental Figure 6D). Since we had sequenced RNAs from the polysome fraction of the sucrose cushion, but the RPF size did not meet expectations, these data were only used for analysis of transcript-level information (TE), rather than codon-level information. TE was calculated by dividing the TPM in the total RNA library by the TPM in the ribosome-bound library for each individual transcript and sample. Differential gene expression for the RNA-Seq was determined using DESeq2 (Bioconductor). Pathway enrichment analysis was performed using mouse gene symbols orthology-mapped to the human genome and tested against GOBP gene sets with the Molecular Signatures Database.

### m^6^A-seq.

Three wild-type C57BL/6J mice (JAX 000664, 2 male 1 female) aged 8 weeks were used. Crypts were isolated from the distal half of the small intestine as described above and dissociated in 10% FBS in 1× PBS supplemented with 20 μg/mL Liberase TH (Roche 05401135001) and 35 μg/mL DNaseI (Roche 10104159001) for 20 minutes at 37°C with frequent agitation. Cell suspensions were then stained with 1:200 PE-EpCAM (BioLegend 118205), 1:200 FITC-CD45 (BioLegend 103107), and 1:1,000 DRAQ7 (Thermo Fisher Scientific D15106) on ice for 30 minutes in the dark. Approximately 800,000 live epithelial cells per mouse were then isolated by flow cytometry by sorting for DRAQ7^–^CD45^–^PE^+^ cells directly into Trizol LS (Ambion 10-296-010) on a FACSJazz Sorter. RNA was isolated from Trizol LS using the Direct-zol RNA Microprep Kit (Zymo R2062). RIP-Seq was performed according to the “Refined RIP-seq” protocol for low input material (42) with the following specifications: 2 μg anti-m^6^A antibody (Synaptic Systems 202 003) was conjugated to magnetic beads and incubated with 6 μg total RNA that was previously fragmented to approximately 300 nt fragments with 10 mM ZnCl_2_ in 10 mM Tris-HCl for 4 minutes at 70°C (5% of fragmented RNA was set aside as input). After 2 hours of immunoprecipitation at 4°C, RNA-bead complexes were washed in high- and low-salt buffer, and RNA was eluted from the RNA-bead complexes using the RNeasy Plus Micro Kit (QIAGEN 74034). Isolated m^6^A-enriched RNA was immunoprecipitated a second time using 2 μg of a second anti-m^6^A antibody (Abcam ab151230). Final cDNA Libraries were prepared from twice immunoprecipitated RNA and fragmented input total RNA using the Smarter Stranded Total RNA-Seq Kit v2 - Pico Input Mammalian (Takara Bio 634411) and sequenced on a NovaSeq 6000, SP Reagent Kit 1.5 (100 cycles). Raw reads were aligned to mm10 (gencode_M23_GRCm38.p6) using STAR aligner (2.7.9a). m^6^A peaks were identified using exomePeak2 (version 1.2.0), followed by a metagene analysis using the Bioconducter packages Guitar_2.10.0 and TxDb.Mmusculus.UCSC.mm10.knownGene_3.10.0.

### m^6^A-RIP-qPCR.

For m^6^A-RIP-qPCR, 4 *Mettl3^fl/fl^* and 4 *Mettl3*^VilCreERΔ*/*Δ^ mice aged 8 weeks were used (2 male, 2 female mice per genotype). Intestinal crypts were isolated from the distal half of the small intestine as described above but without digestion to single cells. Whole crypts were pelleted, then resuspended in TRI Reagent (MilliporeSigma 93289), and RNA was isolated using the Direct-zol RNA Miniprep Kit (Zymo R2050). Next, 20 μg of total RNA was fragmented and immunoprecipitated twice according to the “Refined RIP-seq protocol” as described above. Immunoprecipitated RNA and fragmented input total RNA were reverse-transcribed using the High-Capacity cDNA Reverse Transcription Kit (Thermo Fisher Scientific 4368814), and qPCR was performed as described in the *qRT-PCR* section. For calculation of m^6^A enrichment, first we calculated the %input for every target in every RIP sample as 2^(Ct^ ^of^ ^target^ ^gene^ ^in^ ^RIP^^–^^Ct^ ^of^ ^target^ ^gene^ ^in^ ^input^ ^sample)^. Then we calculated m^6^A enrichment relative to *Gapdh* by dividing the %input of the target of interest (e.g., *Kras* 5′ or 3′UTR) by the %input of *Gapdh* for each sample.

### Lentiviral constructs and transduction.

Accutase-digested (STEMCELL Technologies 07920) enteroids were resuspended in ENR with 1:4 lentivirus solution, TransDux MAX Lentivirus Transduction Enhancer (System Biosciences LV860A-1), and 10 μM Y-27632 and plated as monolayers on Collagen I (Advanced Biomatrix 5010). Lentiviral constructs and prepared virus were generated by VectorBuilder. The transfer vector for catalytic inactive METTL3 expression contained a CMV-EGFP:T2A:Puro selection cassette and the mPGK promoter upstream of the *Mus musculus Mettl3* ORF edited at ORF positions 1183–1194 from GACCCACCTTGG to GCCCCACCTGCG yielding DPPW>APPA synonymous mutation in the catalytic site as previously described (35). The transfer vector for KRAS or KRAS^G12D^ expression contained a CMV-EGFP:T2A:Puro selection cassette and the CMV promoter upstream of the *Mus musculus Kras* ORF either unedited (KRAS OE) or edited at ORF position 35 from G to A yielding G>D synonymous mutation in the regulatory site as previously described (KRAS^G12D^ OE) (61). The “GFP” control vector contained a CMV-mCherry:T2A:Puro selection cassette and the EGFP ORF under control of the CMV promoter. After overnight incubation with lentivirus, enteroid monolayers were mechanically dissociated and replated as 3D enteroids in Matrigel and selected for antibiotic resistance genes after 48 hours. The transfer vector expression rates (after antibiotic selection) for all transgenic lines were determined by assessing rates of GFP reporter expression using flow cytometry and are given in Supplemental Table 4.

### Statistics.

Unless otherwise noted, quantification of immunofluorescent staining was performed using 3 representative images taken per mouse using a 20× objective. Images were taken in 3 areas of most severe histological distortion (defined as a high-powered field with greatest changes in crypt and/or villus morphology) in distal half of the small intestine of tested mice. Three representative sections were chosen from matching regions of the distal half of the small intestine in control mice. Quantification was performed while blinded to mouse genotype. Unless otherwise noted, each graphed data point is the mean of 3 quantified representative images per mouse. *P* values were calculated with the unpaired parametric 2-tailed Student’s *t* test in GraphPad Prism. *P* values for survival curves were calculated using the log-rank Mantel-Cox test in GraphPad Prism. Unless otherwise noted, *P* values less than 0.05 were considered significant.

### Study approval.

Mouse experiments and handling were approved under IACUC protocol 001278 at the Children’s Hospital of Philadelphia.

### Data availability.

All data needed to evaluate the conclusions in the paper are present in the paper or the supplemental materials. See complete unedited blots in the supplemental material. Values for all data points in graphs are reported in the Supporting Data Values file. Raw sequencing data are uploaded to Dryad at https://doi.org/doi:10.5061/dryad.5tb2rbp8s

## Author contributions

CHD and KE Hamilton conceived the study. CHD, EAM, SV, and PS developed methodology. CHD, KE Hayer, SV, YZ, and BJW performed formal analysis. CHD, KEN, SV, RM, SKN, KK, LRP, XM, and AC investigated. CHD and KE Hayer visualized data. CHD, KE Hamilton, PS, and MDW supervised. CHD and KE Hamilton wrote the original draft. CHD, KE Hamilton, MDW, KEN, and KK reviewed and edited the manuscript.

## Supplementary Material

Supplemental data

Supplemental data set 1

Supporting data values

## Version 1 10/26/2023

In-Press Preview

## Version 2 12/08/2023

Electronic publication
