# Supplementary material for: Intestinal transit-amplifying cells require METTL3 for growth factor signaling and cell survival
Source: JCI Insight. 2023 Dec 8;8(23):e171657. doi: 10.1172/jci.insight.171657 (PMC10795831; doi:10.1172/jci.insight.171657)

Supplementary Materials for

Intestinal transit amplifying cells require METTL3 for growth factor signaling and cell survival

Charles H. Danan et al.

*Corresponding author. Email: hamiltonk1@chop.edu

This PDF file includes:

Supplemental Figures 1-9
Supplemental Tables 1-4

Other Supplementary Materials for this manuscript include the following:

Supplemental Data 1-3

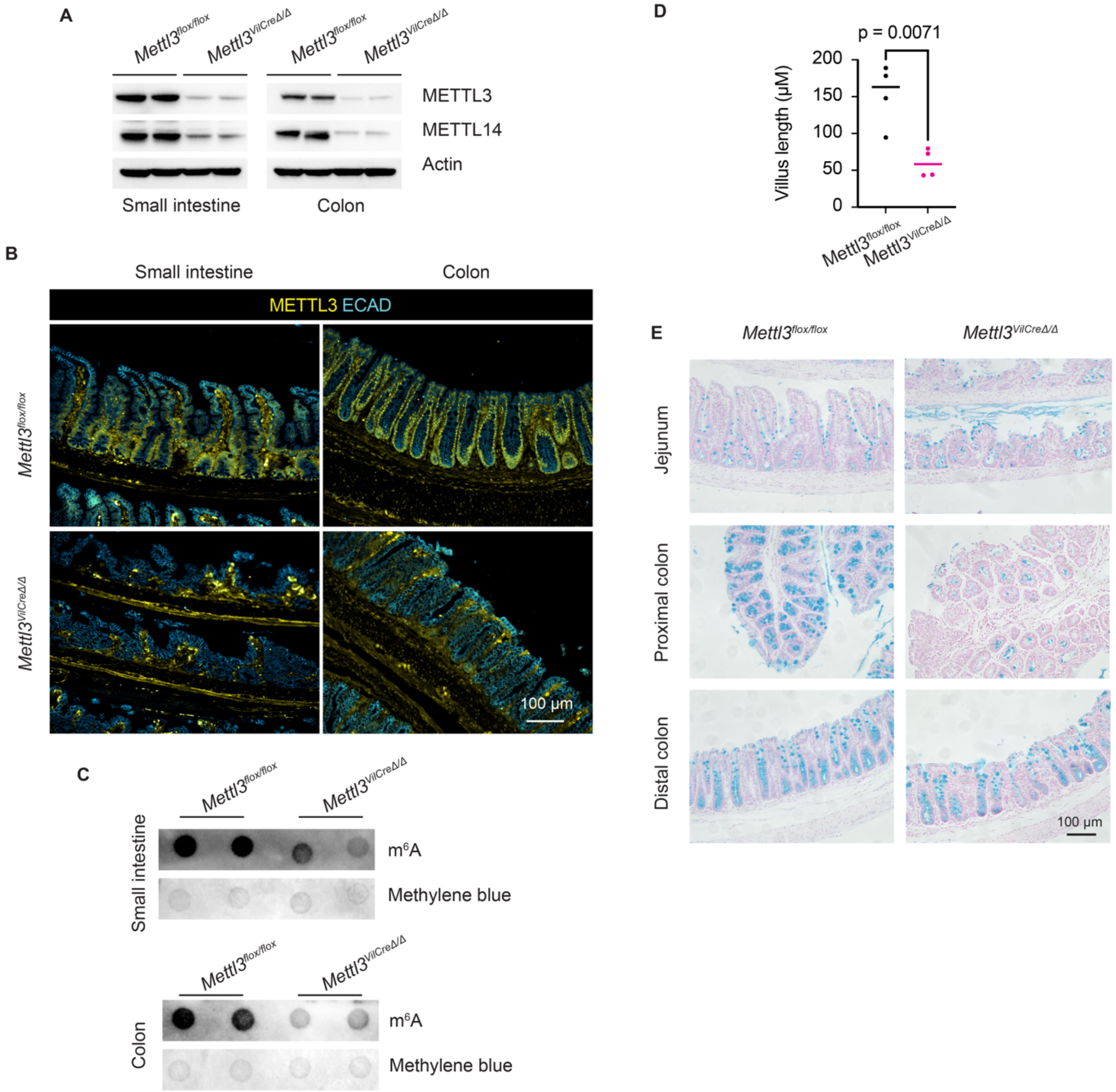

Supplemental Figure 1. Validation and additional characterization of *Mettl3*$^{VilCre\Delta/\Delta}$ mice. (A) Western blot for METTL3 and METTL14 in epithelial crypt enriched lysates from distal half of small intestine and colon. **(B)** Immunofluorescent staining of METTL3 in jejunum. **(C)** m^{6}A dot blot in isolated crypts of distal half of small intestine and colon. **(D)** Quantification of shortest villus lengths in three representative jejunal sections in *Mettl3*$^{flox/flox}$ (n=4) and *Mettl3*$^{VilCre\Delta/\Delta}$ (n=4) mice. Bar at median value. P denotes value of unpaired parametric Student's t test. **(E)** Representative Alcian blue staining in small intestine and colon. All data from postnatal day 29.

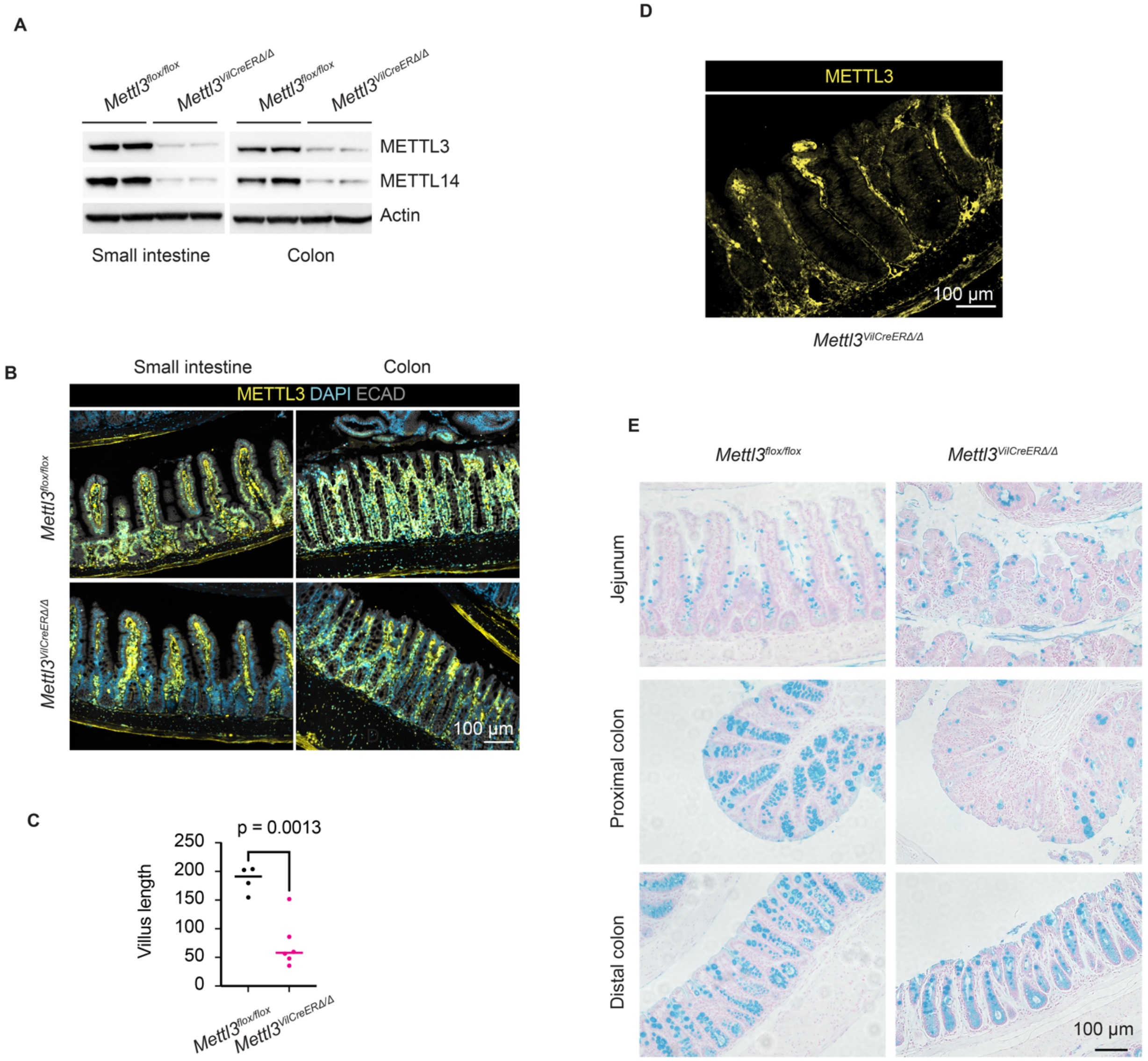

Supplemental Figure 2. Validation and additional characterization of inducible *Mettl3*$^{VilCreER\Delta/\Delta}$ mice. (A) Western blot for METTL3 and METTL14 in epithelial crypt enriched lysates from distal half of small intestine and colon in mice two days post final tamoxifen injection. **(B)** Immunofluorescent staining of METTL3 in jejunum and colon two days post final tamoxifen injection. **(C)** Quantification of shortest villus lengths in three representative jejunal sections in *Mettl3*$^{flox/flox}$ (n=4) and *Mettl3*$^{VilCreER\Delta/\Delta}$ (n=6) mice. Bar at median value. P denotes value of unpaired parametric Student's t test. **(D)** METTL3 staining in hypertrophic small intestinal crypts in a *Mettl3*$^{VilCreER\Delta/\Delta}$ mouse nine days post final tamoxifen injection. **(E)** Representative Alcian blue staining nine days post final tamoxifen injection.

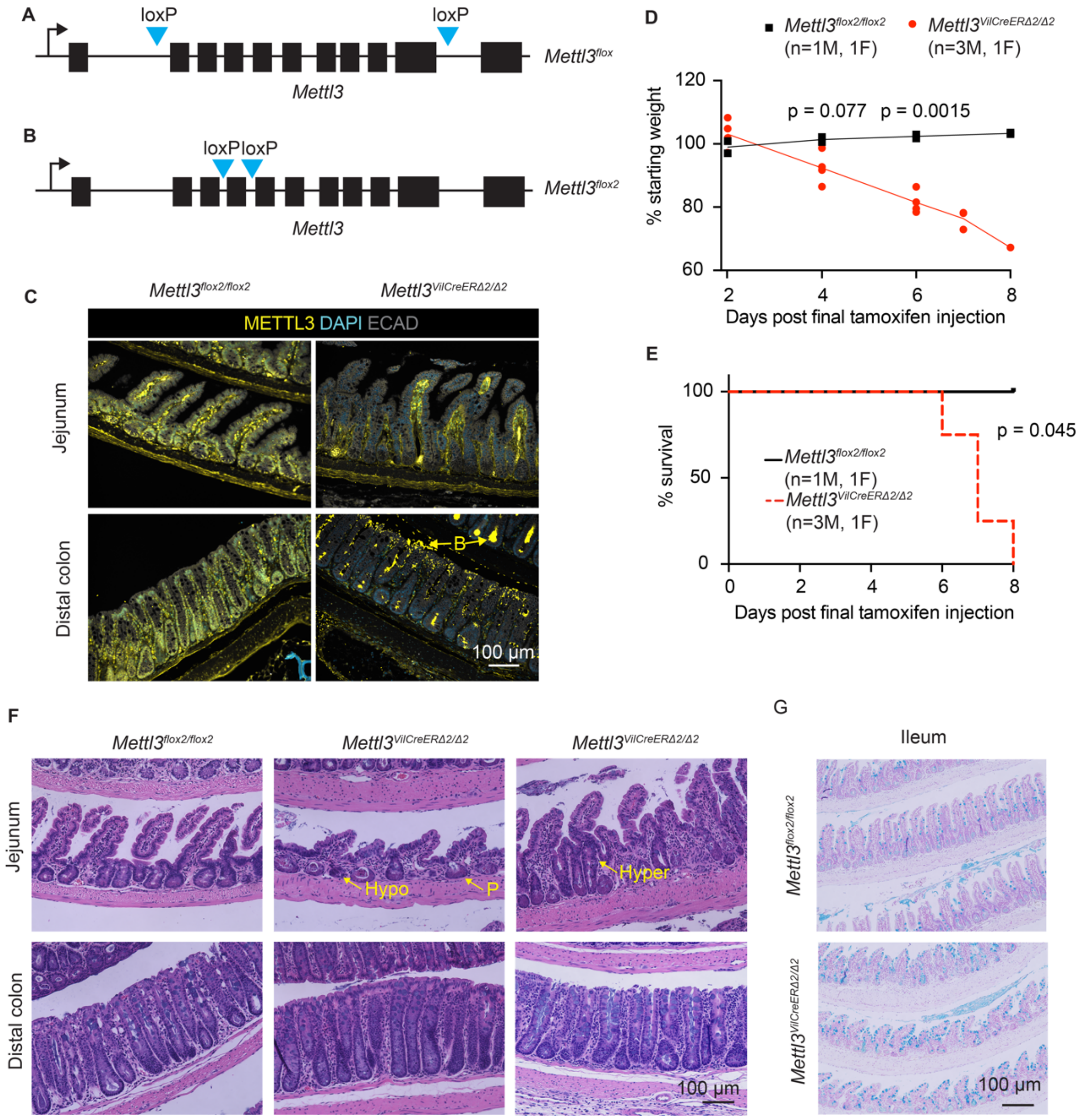

Supplemental Figure 3. A second *Mettl3* deletion model recapitulates intestinal phenotypes. (A, B) Schematic depicting *loxP* sites in the *Mettl3* gene. **(C)** METTL3 staining in jejunum and distal colon. “B” indicates presumed off-target luminal bacterial staining with anti-METTL3 antibody. **(D)** Weight loss post final tamoxifen injection in *Mettl3*$^{flox2/flox2}$ (n=2) and *Mettl3*$^{VilCreER\Delta2/\Delta2}$ (n=4) mice. Each individual point represents one mouse. P denotes value of unpaired parametric Student’s t test at days 4 and 6. **(E)** Kaplan-Meier survival curve post final tamoxifen injection in *Mettl3*$^{flox2/flox2}$ (n=2) and *Mettl3*$^{VilCreER\Delta2/\Delta2}$ (n=4) mice. P value corresponds to Log-rank (Mantel-Cox). **(F)** Representative H&E images from small intestine and colon. “Hypo” indicates hypoplastic crypts. “Hyper” indicates hyperplastic crypts. “P” indicates crypts dominated by Paneth cell granules. **(G)** Representative Alcian blue staining of ileum. Images from areas of most severe histological distortion in distal small intestine of mice meeting euthanasia criteria or littermate, tamoxifen-injected controls.

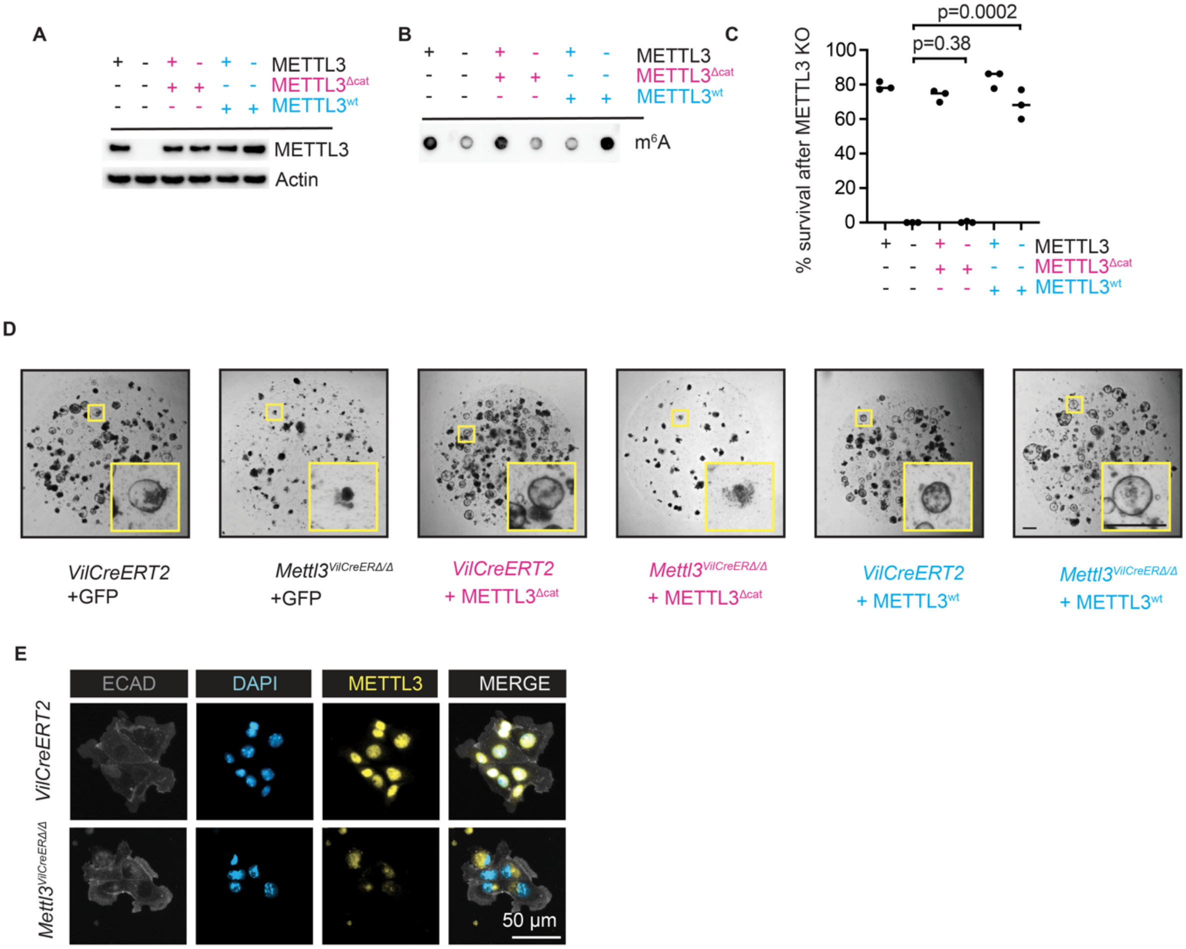

Supplemental Figure 4. Catalytic inactive METTL3 does not rescue death of *Mettl3*$^{VilCreER\Delta/\Delta}$ enteroids. (A) Western for METTL3 two days post 4-OHT in *VilCreERT2* or *Mettl3*$^{VilCreER\Delta/\Delta}$ enteroids infected with lentivirus expressing either GFP, METTL3$^{\Delta cat}$, or METTL3^{wt}. **(B)** m^{6}A dot blot three days post 4-OHT in enteroid lines depicted in (A). Each dot is 60 ng isolated mRNA. **(C)** Percent surviving organoids five days post 4-OHT in enteroid lines depicted in (A). N=3 passage separated biological replicates are plotted with bar at media value, each biological replicate is comprised of three technical replicates. P value represents unpaired parametric Student's t-test **(D)** Representative images of enteroid appearance from data depicted in (C). Representative individual enteroids are highlighted in yellow insets. Scale bar 100 μM. **(E)** Whole mount staining of METTL3 in intestinal epithelial monolayers two days post 4-OHT treatment.

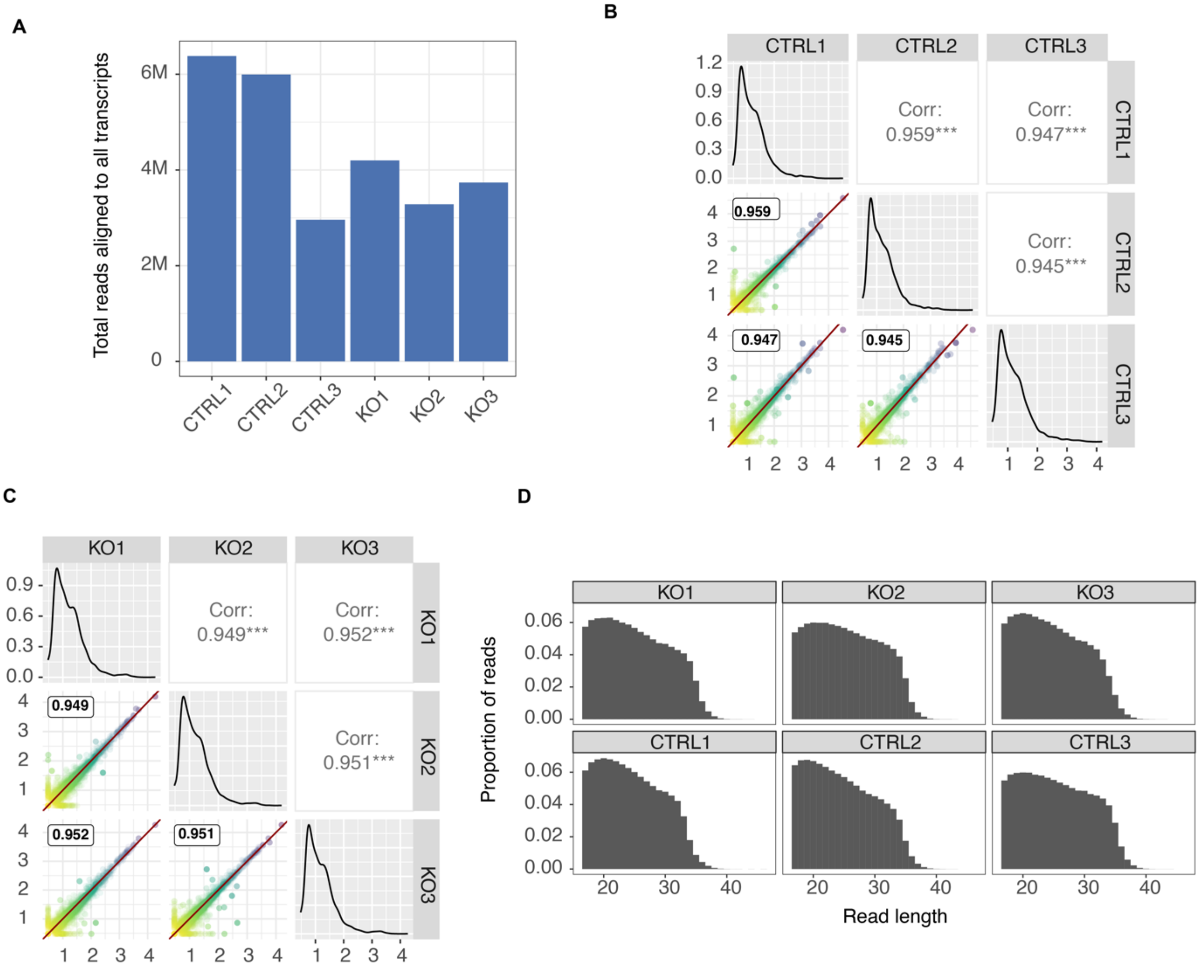

Supplemental Figure 5. Quality control for the polysome sequencing. (A) Read depth for all polysome profiling samples. **(B and C)** Correlation analysis for all (B) control samples and (C) METTL3 KO samples (C). **(D)** Distribution of read length for all polysome profiling samples.

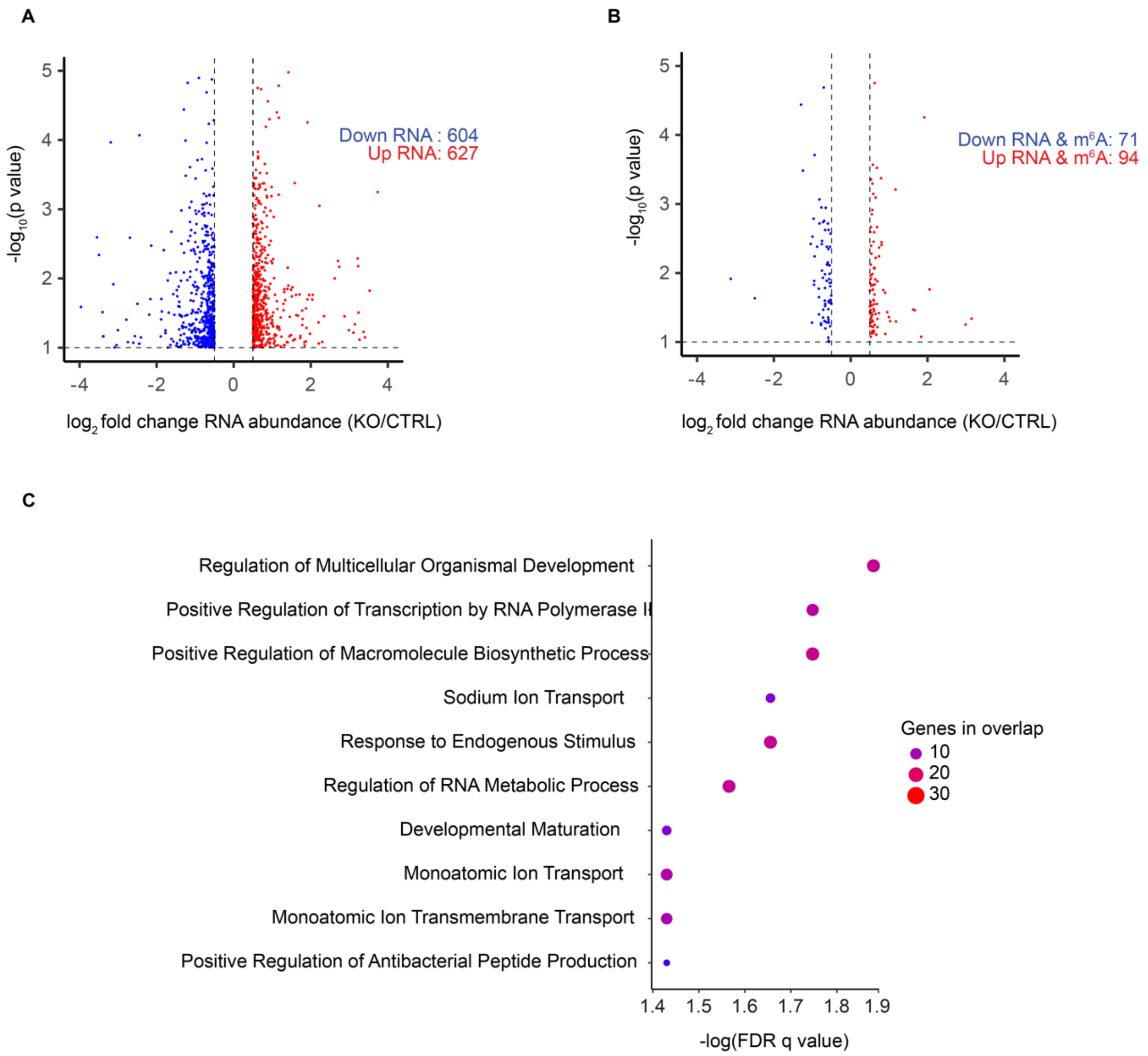

Supplemental Figure 6. Minimal changes in RNA abundance immediately following METTL3 deletion. (A) Volcano plot of all transcripts with log_2 fold change in RNA abundance >0.5 or <-0.5 and -log_{10} p value >1. Data for transcripts detected in RNA-seq of $Mettl3^{flox/flox}$ (CTRL) and $Mettl3^{VilCreER\Delta/\Delta}$ (KO) ileal enteroids 72 hours post initiation of 4-OHT treatment. Red marks all transcripts with significantly increased RNA abundance and blue marks all transcripts with significantly decreased RNA abundance. **(B)** Volcano plot of all transcripts displayed in (A), now filtered for transcripts containing at least one m^{6}A peak. **(C)** Pathway enrichment analysis comparing transcripts with upregulated RNA abundance (log_2FC < -1) and at least one m^{6}A peak against Gene Ontology Biological Process (GOBP) gene sets. Circle color and size both scale with number of genes overlapping between the tested gene set and the GOBP gene set.

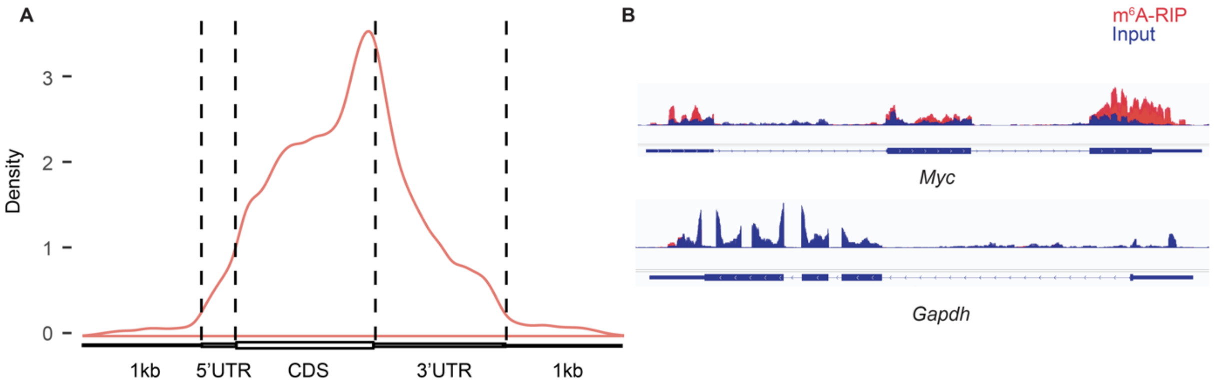

Supplemental Figure 7. Quality control for m^{6}A-seq. (A) Metagene density plot depicting distribution of m^{6}A peaks called by Exomepeak2 within m^{6}A-seq data from wildtype mouse small intestinal crypt epithelium. **(B)** m^{6}A-seq read density (red) compared to input RNA read density (blue) for positive control (*Myc)* and negative control (*Gapdh*) transcripts as seen in Integrated Genomics Viewer.

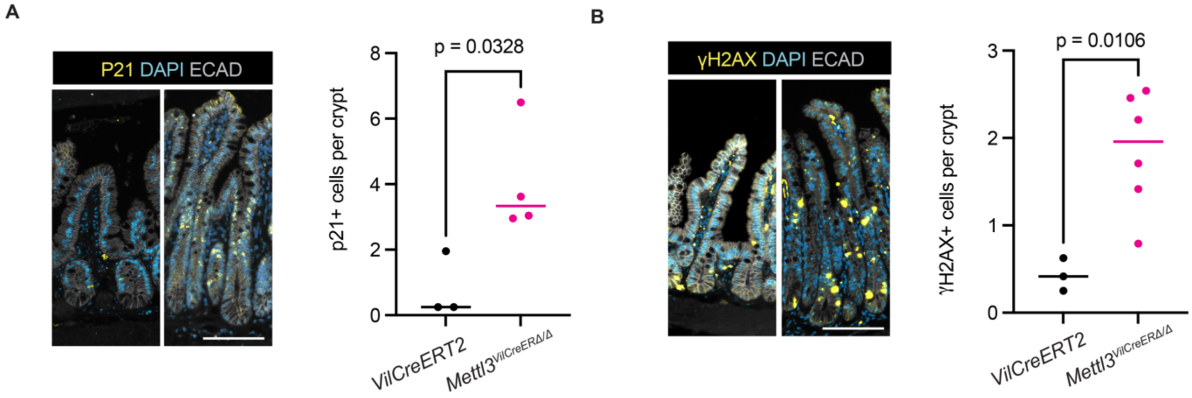

Supplemental Figure 8. Increases in senescence after METTL3 deletion are significant compared to *Villin-CreERT2* controls. (A and B) Representative images and quantification of p21 (n=3,4) and γH2AX (n=3, 6) staining in distal half small intestine of *Villin-CreERT2* and $Mettl3^{VilCreER\Delta/\Delta}$ mice two days post final tamoxifen injection. Values for $Mettl3^{VilCreER\Delta/\Delta}$ mice are the same used in Figure 7, I and J. Images and quantification from areas of most severe histological distortion in distal small intestine of mice two days post final tamoxifen injection. Each data point is the mean of three representative sections imaged per mouse with bar at median value and p denotes value of unpaired parametric Student's t test. Scale bars 100 μM.

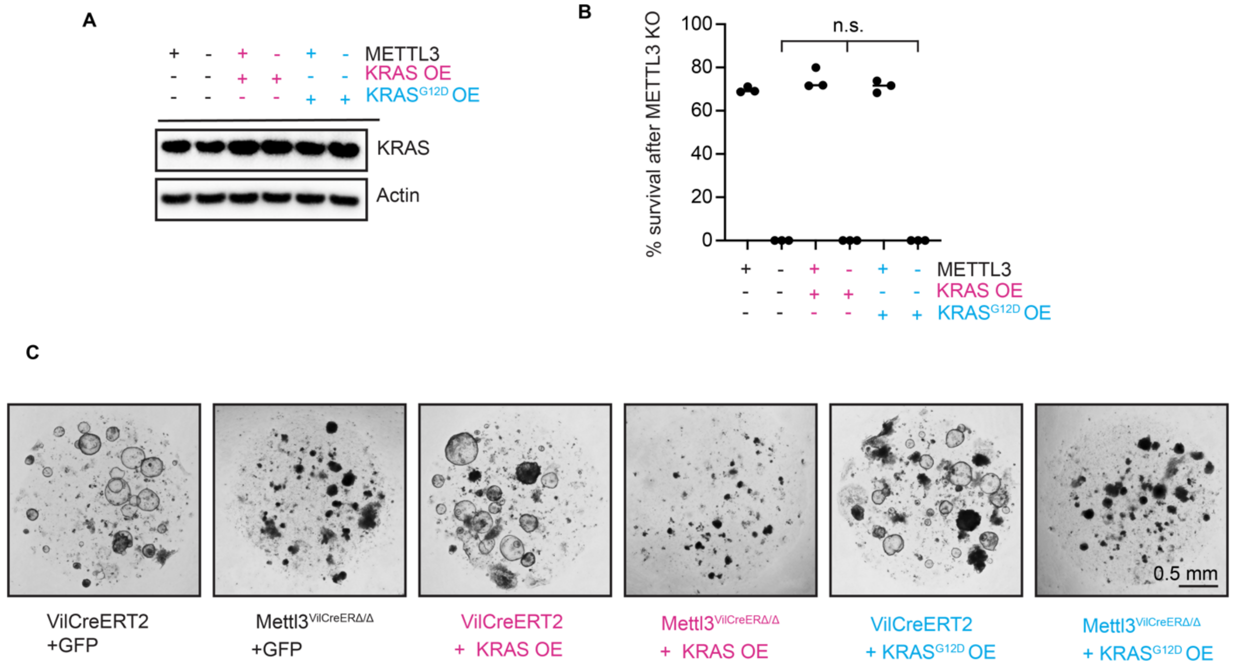

Supplemental Figure 9. KRAS overexpression does not rescue METTL3 KO enteroids. (A) Western for KRAS in tamoxifen-naive enteroids infected with lentivirus expressing either GFP, KRAS, or $KRAS^{G12D}$. **(B)** Percent surviving organoids five days post 4-OHT in enteroid lines depicted in (A). For all quantification, n=3 passage separated biological replicates are plotted with bar at media value, each biological replicate is comprised of 3 technical replicates. Non-significant findings determined using unpaired parametric Student's t-test, represents comparison between METTL3 KO enteroids and METTL3 KO enteroids expressing KRAS or $KRAS^{G12D}$. **(C)** Representative images of enteroid appearance from data depicted in (B)

Supplemental Table 1. Full list of primary and secondary antibodies used in this study.

**Primary antibody- IF**	**Catalog number**	**Antigen retrieval condition (if applicable)**
Rabbit anti-METTL3	abcam ab195352	Tris-EDTA pH 9.0
Rabbit anti-p21	Proteintech 28248-1-AP	Tris-EDTA pH 9.0
Rabbit anti-Phosphorylated-Histone H2A.X (Ser139)	Cell Signaling 9718	Tris-EDTA pH 9.0
Rabbit anti-SP-1 Chromogranin A	Immunostar 20085	Tris-EDTA pH 9.0
Rabbit anti-LYZ1	Dako A0099	Citric Acid pH 6.0
Rabbit anti-MUC2	Genetex GTX100664	Citric Acid pH 6.0
Rabbit anti-Ki67	abcam ab16667	Citric Acid pH 6.0
Rabbit anti-p-ERK	Cell Signaling 4370	Tris-EDTA pH 9.0
Goat anti-E-Cadherin	R&D Systems AF748	Either antigen retrieval method
Mouse anti-beta-Actin	Sigma A5316	
Rabbit anti-METTL14	Sigma HPA038002	
Rabbit anti-UBE1	Proteintech 15912-1-AP	
Rabbit anti-SEC13	Proteintech 15397-1-AP	
Mouse anti-c-Yes	Santa Cruz sc-8403	
Rabbit anti-KRAS	Proteintech 12063-1-AP	
**Secondary antibody- IF**		
Alexa Fluor 488 Anti-goat IgG	Jackson 805-545-180	
Cy3 Anti-Rabbit IgG	Jackson 711-165-152	
**Secondary antibody- Western**		
Anti-rabbit HRP	Cell Signaling 7074	
Anti-mouse HRP	Novus NBP1-75249	

Supplemental Table 2. Histopathological scoring rubric adapted from (56) used for small intestine and colonic histopathological scoring, with scoring rules added for villus damage (villus damage only assessed in small intestine).

Feature graded	Grade	Description
Inflammation	0	None
	1	Slight
	2	Moderate
	3	Severe
Extent	0	None
	1	Mucosa
	2	Mucosa and submucosa
	3	Transmural
Regeneration	4	No tissue repair
	3	Surface epithelium not intact
	2	Regeneration with crypt depletion
	1	Almost complete regeneration
	0	Complete regeneration or normal tissue
Crypt damage	0	None
	1	Basal 1/3 damaged
	2	Basal 2/3 damaged
	3	Only surface epithelium intact
	4	Entire crypt and epithelium lost
Villus damage (SI only)	0	No change in villus height
	1	25% reduction in villus height
	2	50% reduction in villus height
	3	75% reduction in villus height
	4	Complete loss of villus
Percent involvement	1	1-25%
	2	26-50%
	3	51-75%
	4	76-100%

Supplemental Table 3. Full list of Taqman qPCR primers used in this study.

**Gene**	**Assay number**
Actb	Mm02619580_g1
Olfm4	Mm01320260_m1
Bmi1	Mm03053308_g1
Clu	Mm01197002_m1
Lgr5	Mm00438890_m1
Ascl2	Mm01268891_g1
Cd44	Mm01277161_m1
Hopx	Mm00558630_m1
Kras 3' UTR (also used for total transcript)	Mm00517494_m1
Kras CDS exons 1-2	Mm00517492_m1
Kras CDS exons 3-4A	Mm01255197_m1
Kras 5' UTR	Mm00517491_m1
Gapdh	Mm99999915_g1

Supplemental Table 4. Lentiviral transfer vector expression rates in all transgenic enteroid lines. All lines expressed GFP reporter to assess lentiviral infection rates. Expression rates determined by gating for DAPI- GFP+ single cells using flow cytometry.

Vector	% GFP+ cells
VilCreERT2 + GFP	39.8%
Mettl3$^{VilCreER\Delta/\Delta}$ + GFP	69.8%
VilCreERT2 + METTL3$^{\Delta cat}$	21.8%
Mettl3$^{VilCreER\Delta/\Delta}$ + METTL3$^{\Delta cat}$	34.8%
VilCreERT2 + METTL3^{wt}	32.8%
Mettl3$^{VilCreER\Delta/\Delta}$ + METTL3^{wt}	60.3%
VilCreERT2 + KRAS	47.5%
Mettl3$^{VilCreER\Delta/\Delta}$ + KRAS	39.7%
VilCreERT2 + KRAS^{G12D}	32.6%
Mettl3$^{VilCreER\Delta/\Delta}$ + KRAS^{G12D}	74.5%

Supplemental Data 1. (separate file). Differential translation efficiency in METTL3 KO enteroids. Full results of RNA-seq and sequencing of polysome-bound RNAs from n=3 *Villin-CreERT2* (CTRL) and n=3 $Mettl3^{VilCreER\Delta/\Delta}$ (KO) enteroid biological replicates 72 hours after initiating 4-OHT treatment. Green columns and blue columns display transcripts per million (TPM) values output by Kallisto in total RNA and polysome-bound RNA fractions, respectively. Light orange columns correspond to translational efficiency (TE) values for each transcript determined by dividing the TPM in the total RNA library by the TPM in the polysome-bound RNA library for each individual transcript and sample. P value refers to the comparison between mean TE of CTRL vs mean TE of KO replicates.

Supplemental Data 2. (separate file). Differential RNA abundance in METTL3 KO enteroids. Results of DESeq2 analysis of RNA-seq from n=3 *Villin-CreERT2* (CTRL) and n=3 $Mettl3^{VilCreER\Delta/\Delta}$ (KO) enteroid biological replicates 72 hours after initiating 4-OHT treatment.

Supplemental Data 3. (separate file). m6A-seq in wildtype mouse crypt epithelium. Full output of exomePeak2 analysis of m^{6}A-sequencing data produced in epithelial cells sorted from distal small intestinal crypts of n=3 adult wildtype mice.

Lanes used in figure

Full unedited blot for Figure 5C

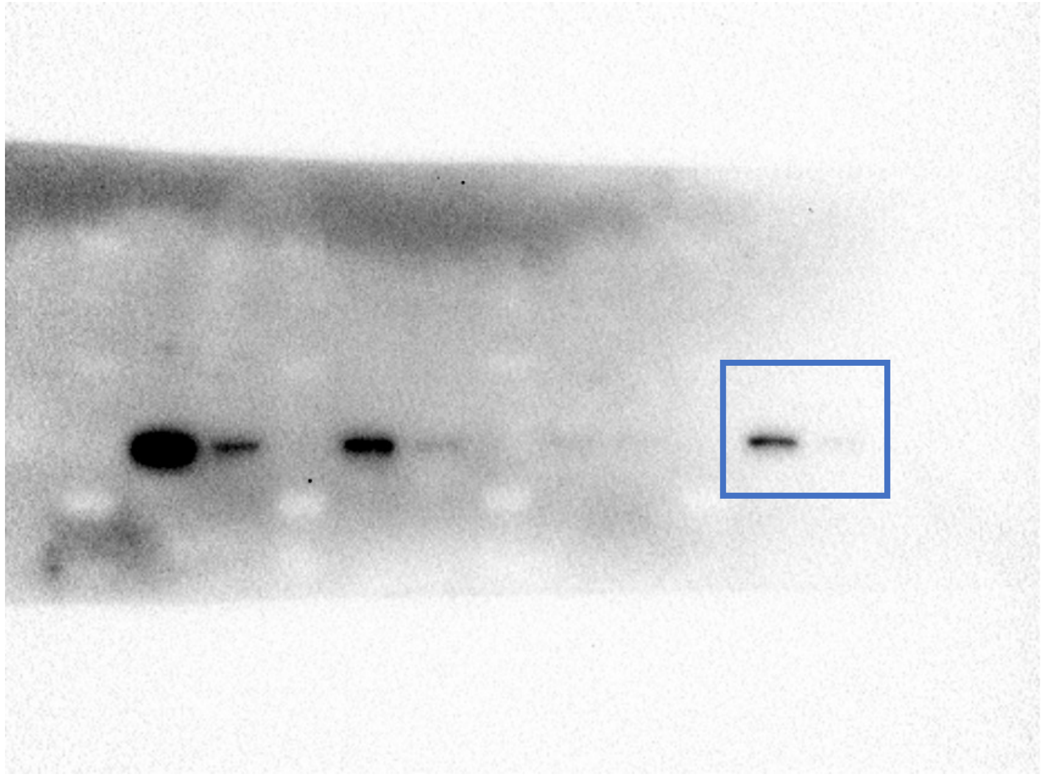

Anti-METTL3

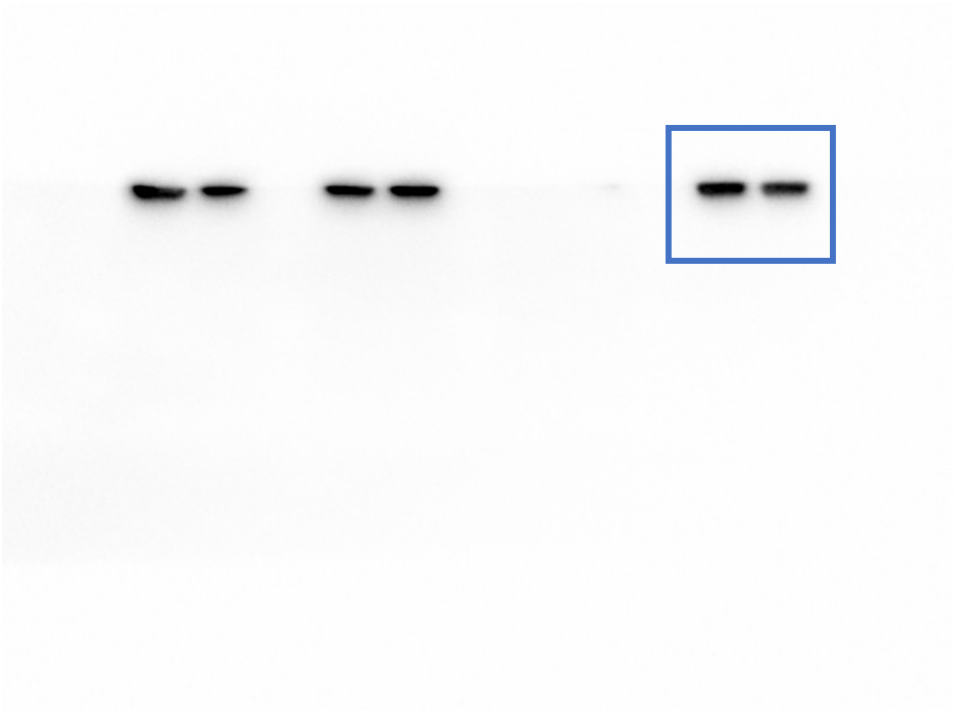

Anti-Actin

Full unedited blot for Figure 7G

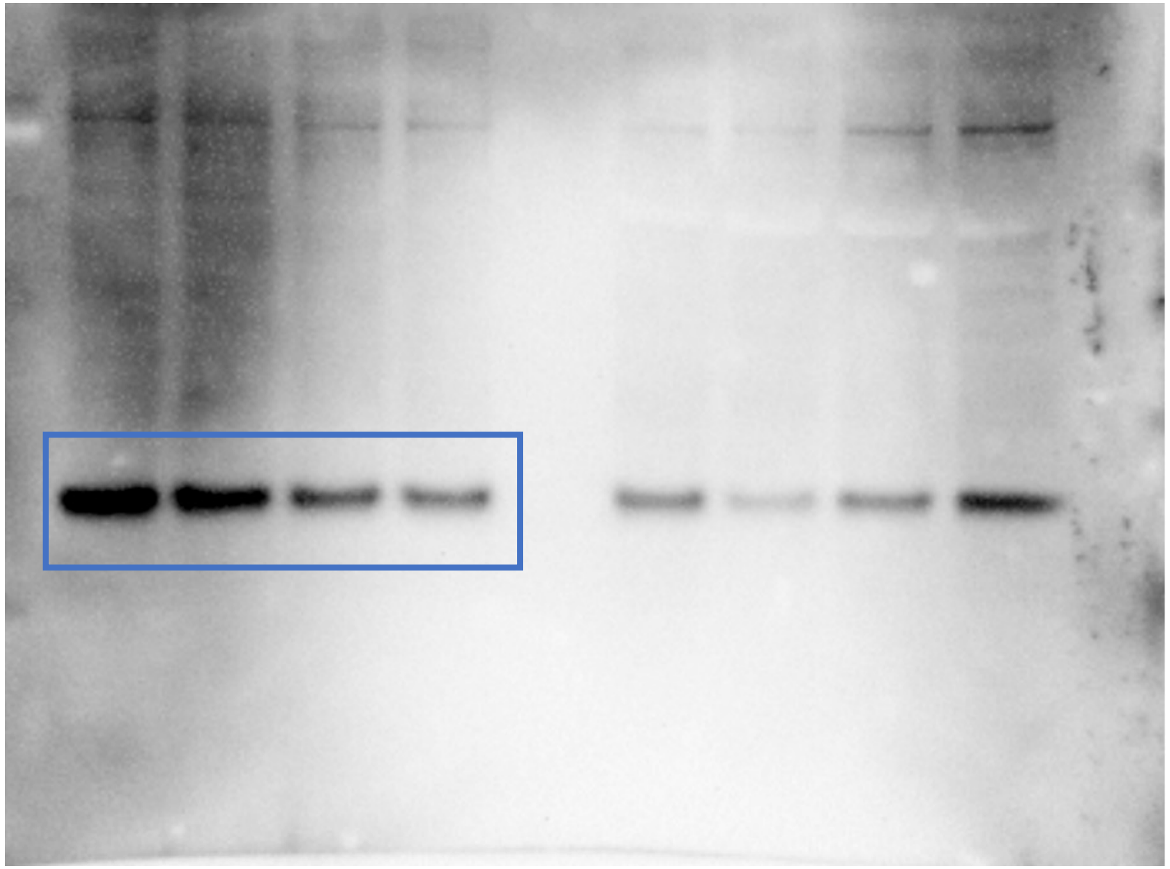

Anti-KRAS

Full unedited blot for Figure 7G

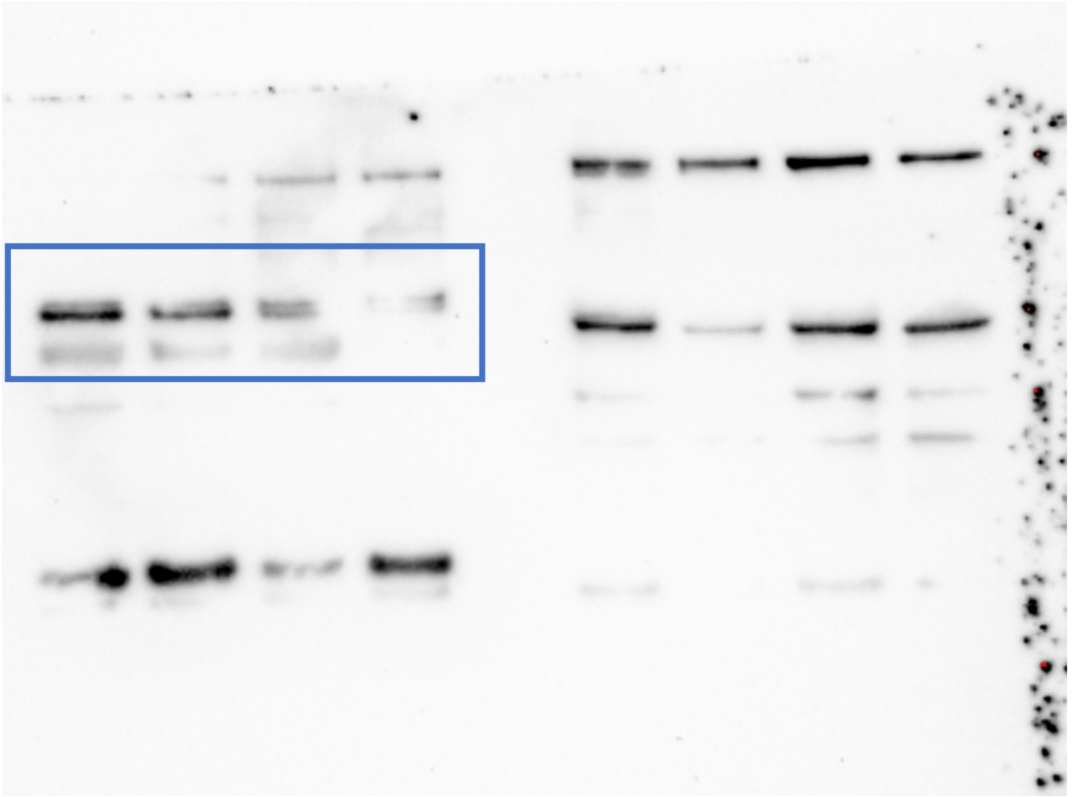

Anti-YES1

Full unedited blot for Figure 7G

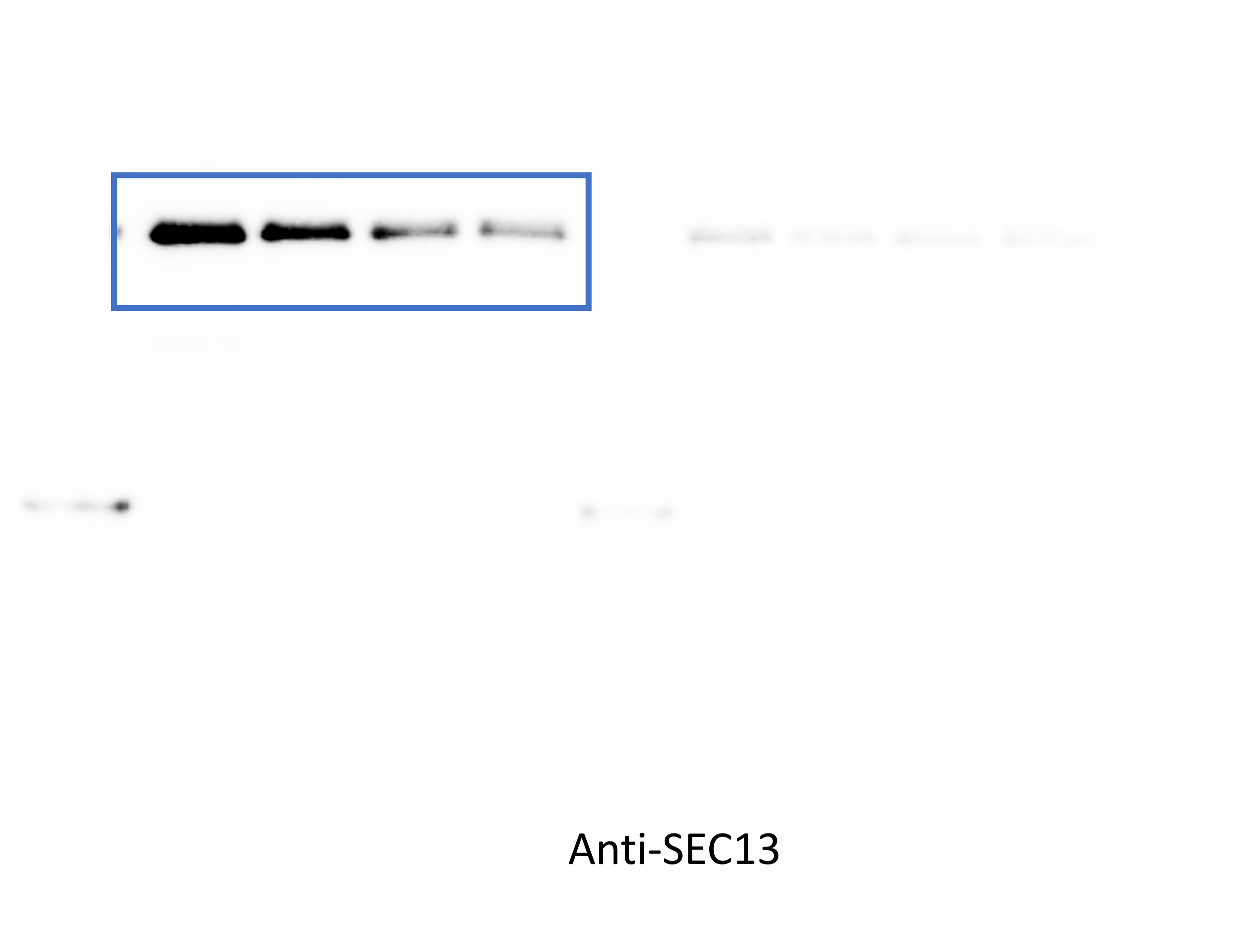

Anti-SEC13

Full unedited blot for Figure 7G

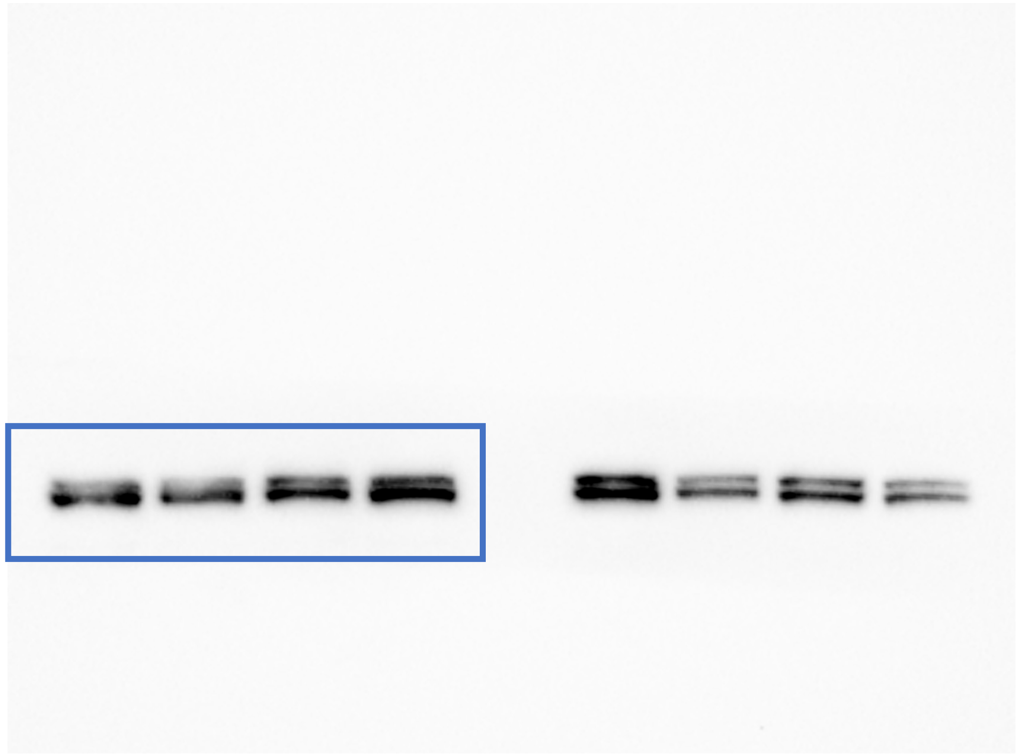

Anti-UBE1

Full unedited blot for Figure 7G

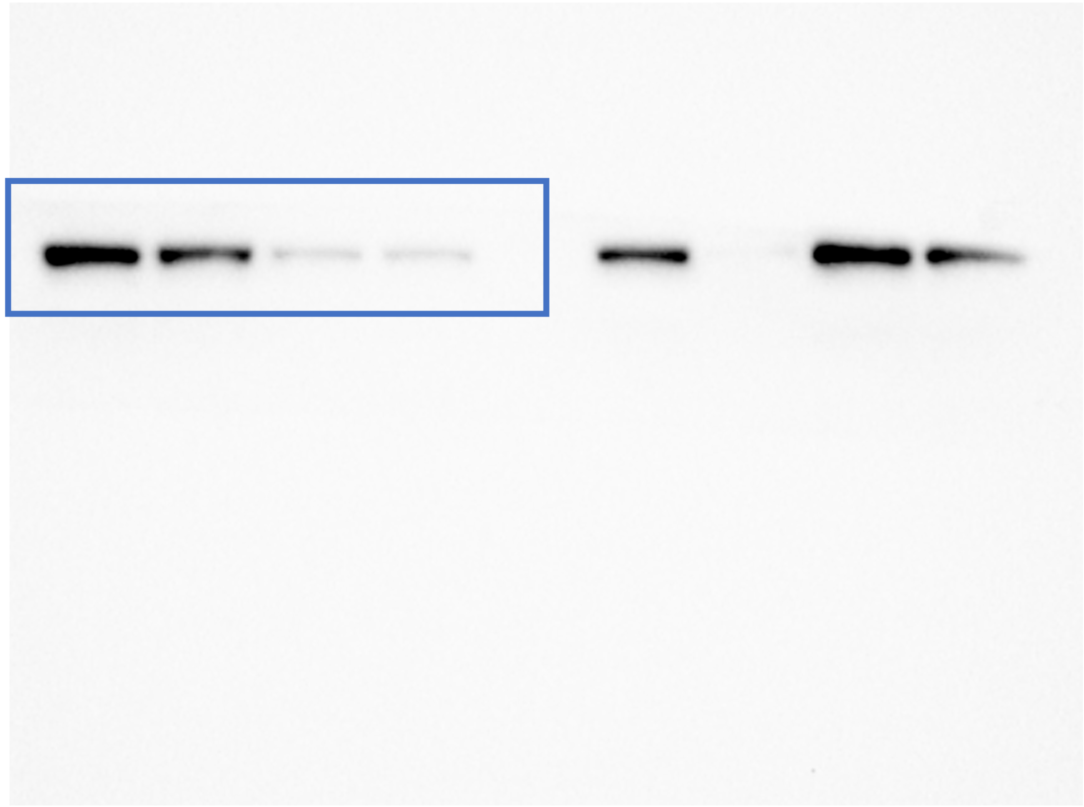

Anti-METTL3

Full unedited blot for Figure 7G

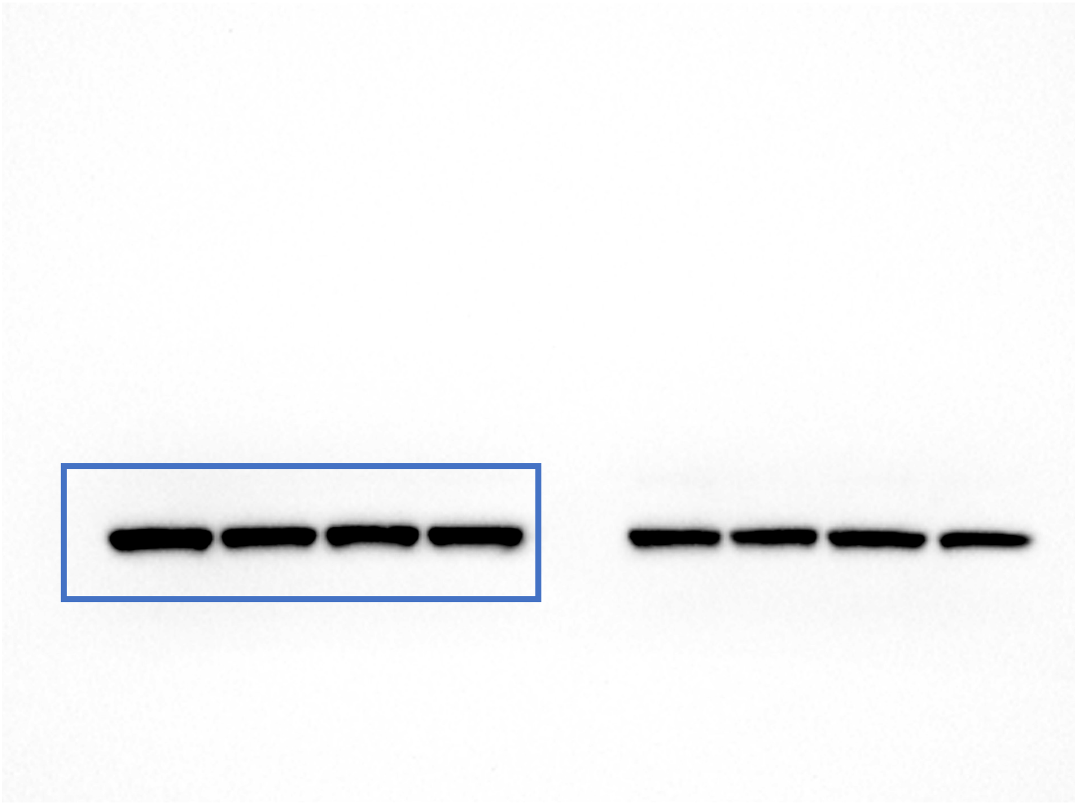

Anti-Actin

Full unedited blot for Supplemental Figure 1A (small intestine)

Anti-METTL3

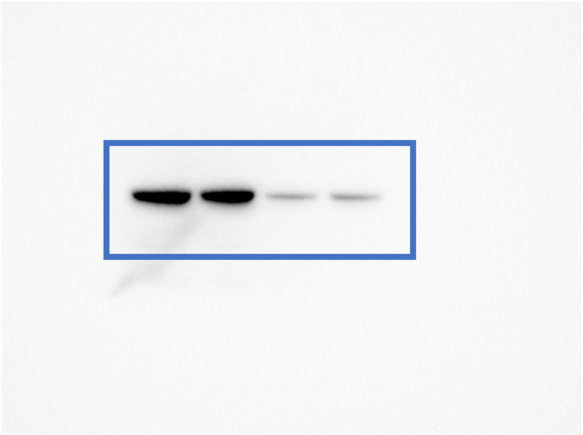

Anti-METTL14

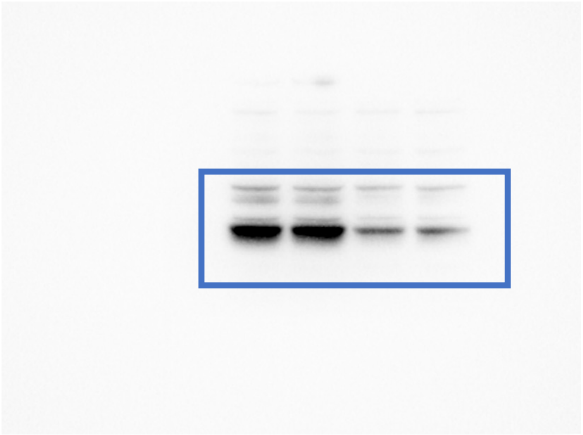

Anti-Actin

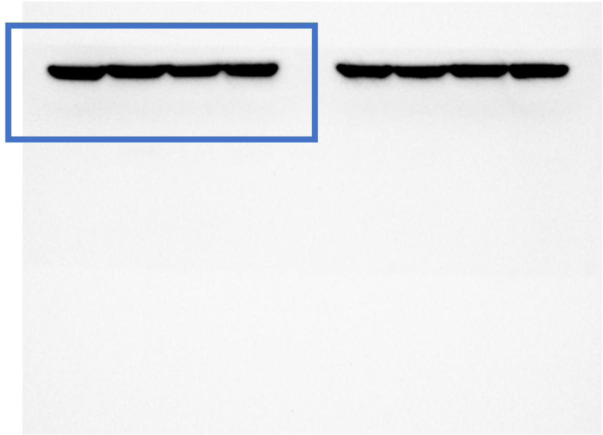

Small intestine

Full unedited blot for Supplemental Figure 1A (colon)

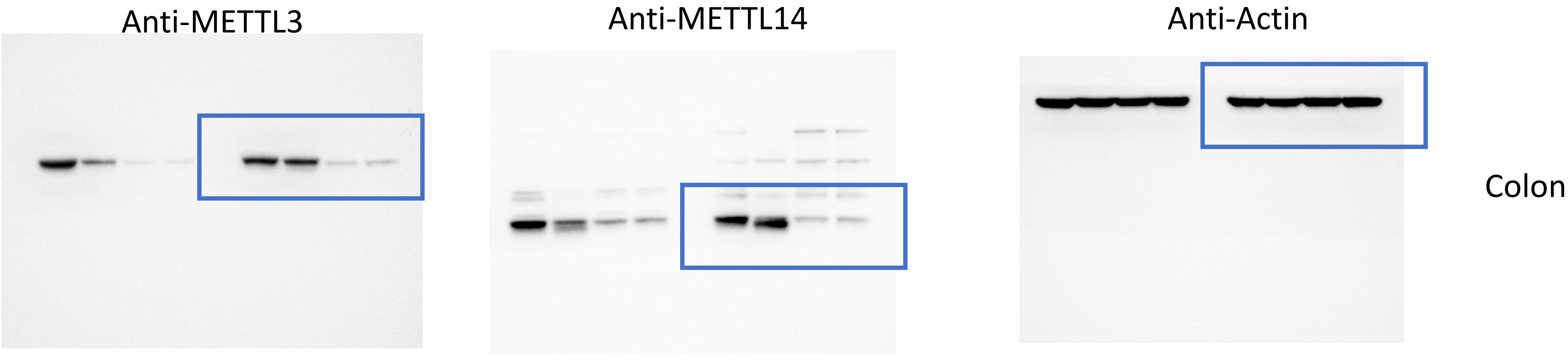

Full unedited blot for Supplemental Figure 1C

m^6A

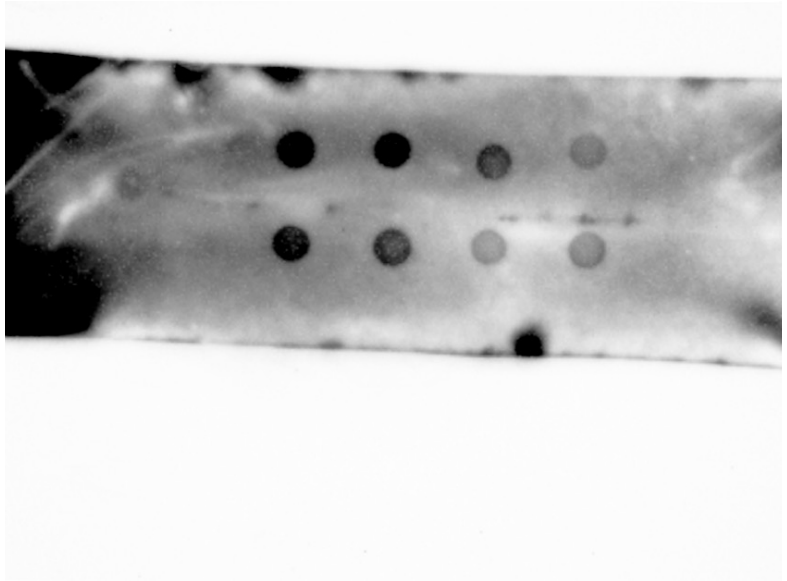

Small intestine

Colon

Methylene blue

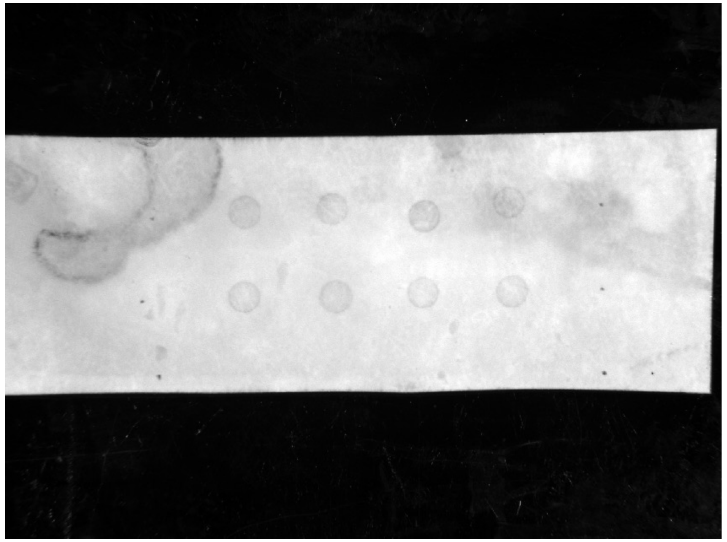

Small intestine

Colon

Full unedited blot for Supplemental Figure 2A

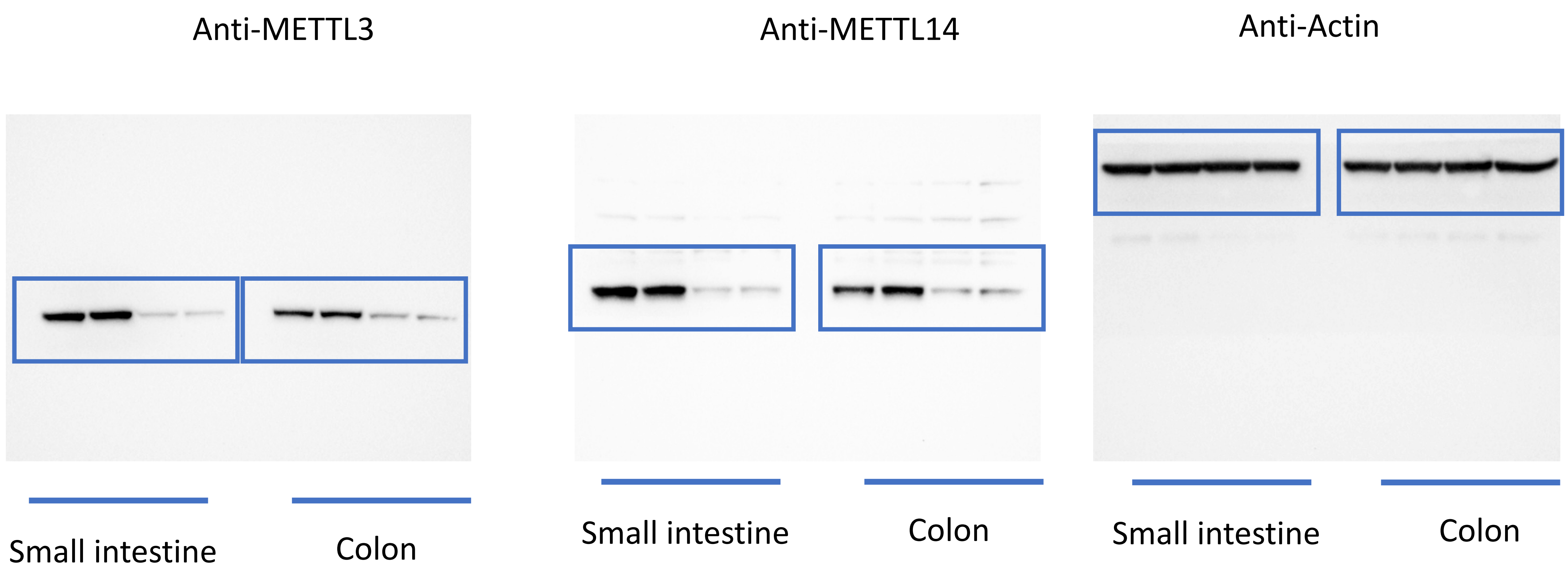

Full unedited blot for Supplemental Figure 4A

Anti-METTL3

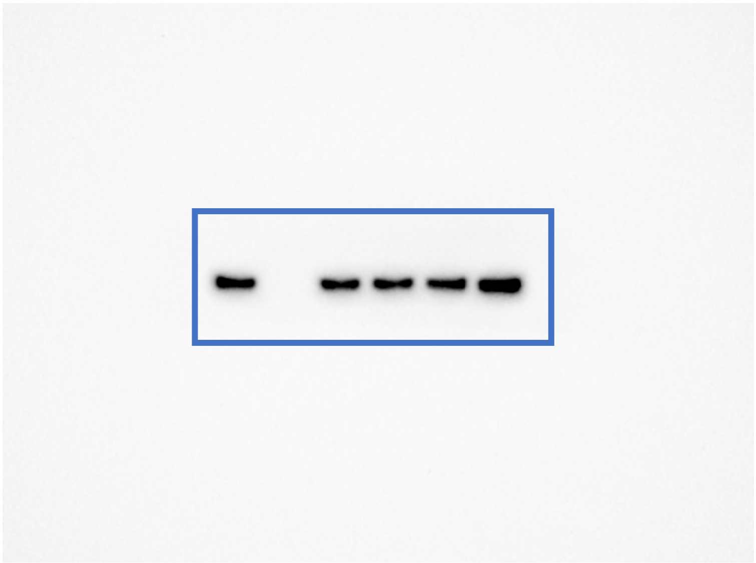

Anti-Actin

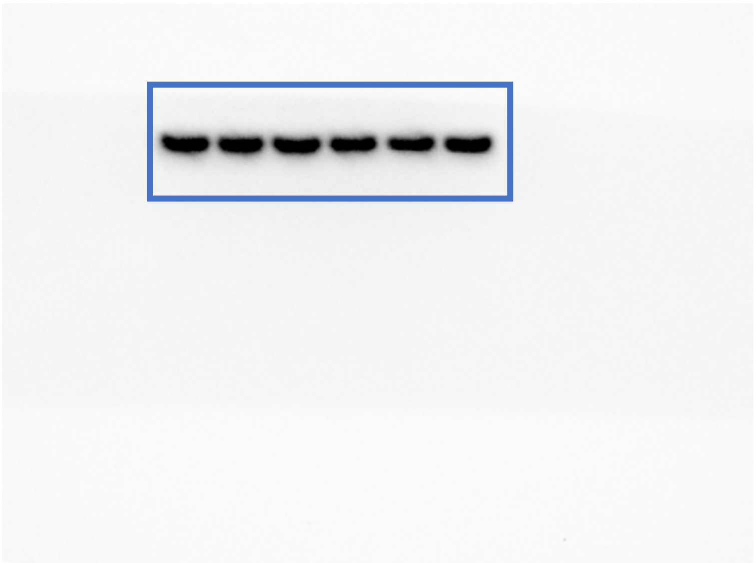

Full unedited blot for Supplemental Figure 4B

Anti-m^{6}A

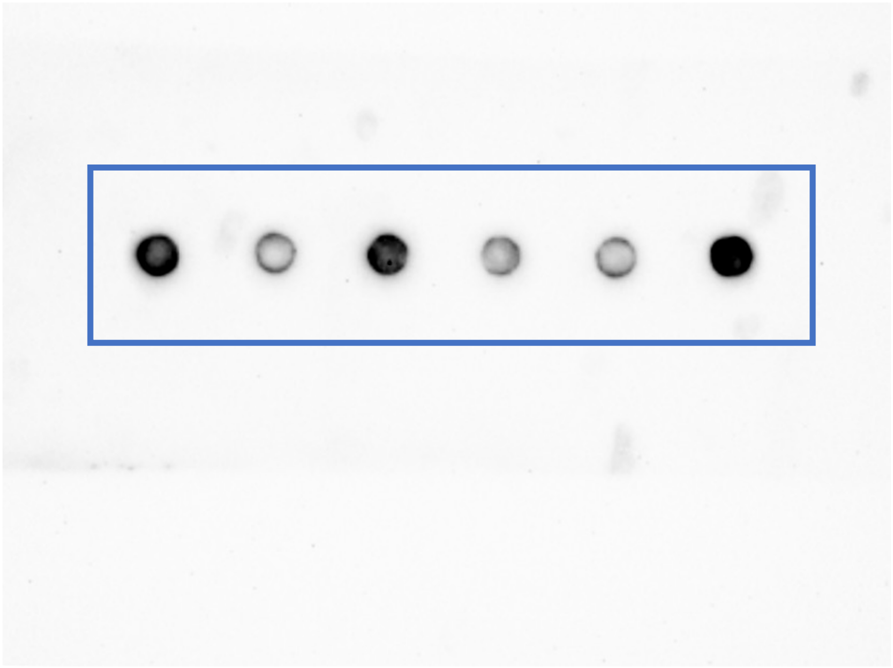

Full unedited blot for Supplemental Figure 9A

Anti-KRAS

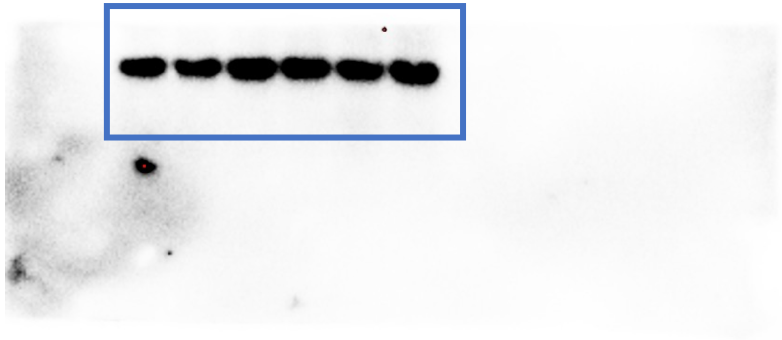

Anti-Actin

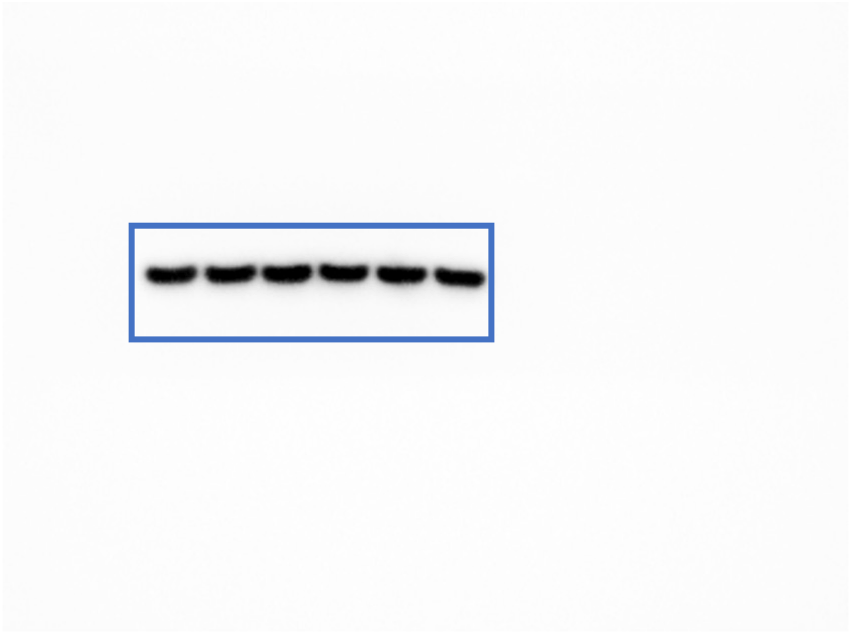

Supplement: Supplemental data [file jciinsight-8-171657-s139.pdf]
